# Loss of H3K9me3 Correlates with ATM Activation and Histone H2AX Phosphorylation Deficiencies in Hutchinson-Gilford Progeria Syndrome

**DOI:** 10.1371/journal.pone.0167454

**Published:** 2016-12-01

**Authors:** Haoyue Zhang, Linlin Sun, Kun Wang, Di Wu, Mason Trappio, Celeste Witting, Kan Cao

**Affiliations:** 1 Department of Cell Biology and Molecular Genetics, University of Maryland, College Park, MD, United States of America; 2 Center for Bioinformatics and Computational Biology, University of Maryland, College Park, MD, United States of America; The University of Hong Kong, HONG KONG

## Abstract

Compelling evidence suggests that defective DNA damage response (DDR) plays a key role in the premature aging phenotypes in Hutchinson-Gilford progeria syndrome (HGPS). Studies document widespread alterations in histone modifications in HGPS cells, especially, the global loss of histone H3 trimethylated on lysine 9 (H3K9me3). In this study, we explore the potential connection(s) between H3K9me3 loss and the impaired DDR in HGPS. When cells are exposed to a DNA-damaging agent Doxorubicin (Dox), double strand breaks (DSBs) are generated that result in the phosphorylation of histone H2A variant H2AX (gammaH2AX) within an hour. We find that the intensities of gammaH2AX foci appear significantly weaker in the G0/G1 phase HGPS cells compared to control cells. This reduction is associated with a delay in the recruitment of essential DDR factors. We further demonstrate that ataxia-telangiectasia mutated (ATM) is responsible for the amplification of gammaH2AX signals at DSBs during G0/G1 phase, and its activation is inhibited in the HGPS cells that display significant loss of H3K9me3. Moreover, methylene (MB) blue treatment, which is known to save heterochromatin loss in HGPS, restores H3K9me3, stimulates ATM activity, increases gammaH2AX signals and rescues deficient DDR. In summary, this study demonstrates an early DDR defect of attenuated gammaH2AX signals in G0/G1 phase HGPS cells and provides a plausible connection between H3K9me3 loss and DDR deficiency.

## Introduction

Hutchinson-Gilford progeria syndrome (HGPS) is a devastating premature aging disorder [1,2]. Patients with HGPS start to develop aging-associated clinical features, including growth retardation, abnormal bone joints, alopecia, and subcutaneous fat loss at the age of 12 to 24-month-old and die at an average age of 13-year-old due to stroke or heart attack [1,2]. HGPS is predominantly (~80%) caused by a *de novo* single point mutation in the exon 11 of the *LMNA* gene (1824 C->T) [3,4]. The mutation activates a cryptic splice donor site and yields a 50 amino acid truncated lamin A mutant protein called “progerin” [3,4]. This internal deletion removes a cleavage site of Zmpste24 (a zinc metallopeptidase STE24 homolog) from lamin A and consequently interferes with lamin A’s post-translational modifications, causing an abnormal retention of a farnesyl tail on the C-terminus of progerin [3,4]. The farnesylated progerin accumulates on the inner nuclear membrane and causes severe nuclear phenotypes including misshapen nuclear morphology, loss of peripheral heterochromatin, histone modification abnormalities, gene transcription alterations, compromised DDR and genome instability [5–8].

Among these phenotypes, defective DDR has been closely associated with genome instability and premature aging [9]. Abnormal DDR has been observed in HGPS patient primary fibroblast cells and MEFs from HGPS animal models [8,10,11]. Specifically, in response to irradiation, the recruitments of DDR players, such as 53BP1 and Rad51, were significantly delayed [8,10,11]. We recently reported a drastic delay in Rad51 recruitment to DSBs in HGPS iPSC-differentiated smooth muscle cells, suggesting that the defective DDR is a universal phenotype associated with multiple HGPS lineages [11]. Moreover, ectopic expression of progerin in HeLa cells also significantly impaired 53BP1 recruitment to DSBs, and a direct inhibitory role of progerin in DDR was suggested [12].

Phosphorylation of the histone H2A variant H2AX at Serine 139 (gammaH2AX) is a crucial histone modification that occurs very rapidly at DSBs [13,14]. As an upstream signal, gammaH2AX plays an essential role in initiating DSB repair [14]. In a previous study, embryonic stem cells from H2AX deficient (H2AX^Δ/Δ^) mice displayed a delayed recruitment of DDR players, elevated sensitivity to ionizing irradiation and compromised genome integrity [15]. Mechanistically, H2AX phosphorylation was believed to recruit several down-stream DDR proteins including NBS1, MDC1, 53BP1 and BRCA1 to the DSB site to fix DSBs [15–17]. Three kinases, ATM,

ATR or DNAPK have been shown to carry out the phosphorylation of H2AX at DSBs [13,18–20]. In addition, ATM kinase can mediate phosphorylation of adjacent H2AX, thereby amplifying gammaH2AX signals and creating a positive feedback loop [13,21,22]. gammaH2AX was also reported to facilitate DSB end joining by anchoring DNA break ends in close proximities and reducing chromosome density [13,14,23–26].

Over the past decade, aberrant histone modifications have been implicated in the DDR deficiencies in HGPS [10,27,28]. It has been proposed that the histone epigenetic abnormalities render a more condensed chromatin structure and create a physical barrier, preventing DDR players from access to DSBs [10,27,28]. Besides physical allowance, some histone modifications may also functionally regulate DDR. Histone H4 acetylated on lysine 16 (H4K16ac) has been shown to directly control the recruitment of BRCA1 and 53BP1, and histone H3 trimethylated on lysine 9 (H3K9me3) was essential to recruit Tip60 and activate ATM upon DNA damage [29–32]. Notably, loss of H3K9me3 is a well-documented hallmark epigenetic phenotype in HGPS. Whether and how the classic “H3K9me3 global loss” phenotype affects DDR, especially gammaH2AX, in HGPS cells remain to be addressed.

In this study, we aim to investigate DSB-induced gammaH2AX signals and the potential crosstalk between this phenotype and the aberrant H3K9me3 loss phenotype in HGPS cells. Here we report a reduction in gammaH2AX signal strength in HGPS fibroblasts upon treatment with Dox, a DSB inducing agent. Interestingly, this phenotype is predominantly observed in G0/G1 phase cells but not in S phase cells. A similar reduction in gammaH2AX response is observed in G0/G1 phase HGPS cells upon treatment with camptothecin (CPT), a different DSB inducing agent. The reduced amplification of gammaH2AX signals consequently leads to a delay in DDR player recruitment and DSB repair. Furthermore, ATM activation is found to be impaired in G0/G1 phase HGPS fibroblasts upon DSBs, leading to reduced gammaH2AX response. Finally, we show evidence supporting that the observed ATM inactivation is closely correlated with the loss of H3K9me3 in HGPS fibroblasts. Attempts to increase H3K9me3 through methylene blue (MB), an antioxidant which has been recently reported to remove progerin from the nuclear rim and rescue heterochromatin loss in HGPS [33], was able to restore H3K9me3, rescue the defects in ATM activation, gammaH2AX signal strength, and DDR in HGPS fibroblasts. These results suggest a novel connection between the two prominent phenotypes (H3K9me3 loss and DDR deficiency) and provide mechanistic insights underlying the complex phenotypes in HGPS cells.

## Materials and Methods

### Cell culture

Primary HGPS human skin fibroblast cells that carry the classic 1824 C->T mutation (HGADFN167) and normal control cells (HGFDFN168, father of HGADFN167) were obtained from the Progeria Research Foundation (PRF) and cultured under conditions as previously described [33]. For passaging, cells were split at a ratio of 1:2 at 95% confluency. Normal and HGPS fibroblasts at passages 13–15, 18–21 and 22–24 were referred to as early, middle and late passage fibroblasts respectively. Unless otherwise noted, all the experiments were performed on cells from passage 15 to passage 21 (early to middle passages). The passage number is exactly matched between normal and HGPS cells in each experiment.

### Drugs

Drugs used in this study are listed as below: Doxorubicin (Dox, Sigma), camptothecin (CPT, Sigma), ATM specific inhibitor (KU55933, Selleck Chem), ATR specific inhibitor (VE-821, Selleck Chem), DNAPK specific inhibitor (NU7441, Selleck Chem) and methylene blue (MB; Acros Organics).

### Antibodies

Antibodies used in western blotting and immunofluorescence analysis were obtained from the following sources: mouse anti-lamin A/C (Mab3211, Millipore, 1:500), mouse anti- gammaH2AX (05–636, Upstate, 1:500), rabbit anti-gammaH2AX (ab11174, Abcam, 1:1000), rabbit anti-H2AX (2595, cell signaling, 1:500), mouse anti-alpha-tubulin (DM1α, Santa Cruz, 1:1000), mouse anti-GAPDH (ab8245, Abcam, 1:5000), mouse anti-BrdU (555627, BD Pharmingen, 1:500), rabbit anti-RIF1 (A300-568A, Bethyl Laboratory, 1:500), rabbit anti-53BP1 (sc-22760, Santa Cruz, 1:500), rabbit anti-ATM (ab32420, Abcam, 1:1000), rabbit anti-pATM (S1981) (ab81292, Abcam, 1:1000), mouse anti-DNAPK (sc-390495, Santa Cruz, 1:500), mouse anti-pDNAPK (T2609) (ab18356, Abcam, 1:500), mouse anti-CHK1 (sc-8408, Santa Cruz, 1:500), rabbit anti-pCHK1(S345) (2341S, Cell Signaling, 1:500), rabbit anti-H3K9me3 (ab8898, Abcam, 1:1000), rabbit anti-HP1alpha (2616S, Cell Signaling, 1:500), rabbit anti-SUV39h1 (A302-127A, Bethyl Laboratory, 1:500).

### G0/G1 phase synchronization & Cell cycle analysis

Passage-matched normal and HGPS fibroblasts were cultured in serum free MEM (Life Technologies) medium, supplemented with 2mM L-Glutamine (Life Technologies), for 24h for G0/G1 phase synchronization. Cell cycle analysis was then performed as previously described to examine the effect of serum starvation[11]. Briefly, normal and HGPS fibroblasts were harvested by TrypLE Express (Life Technologies) and washed with PBS (HyClone). Cell pellets were re-suspended in 0.5ml PBS and then added with 4.5ml 70% (vol/vol) ice-cold ethanol. The mixture was incubated for 10-15min at 4°C, washed with PBS and then re-suspended in 50ul propidium iodide (Invitrogen) staining buffer (50ug/ml propidium iodide and 100μg/ml DNAse-free RNAse in PBS). The mixture was then allowed to incubate for 30min at 37°C. Flow cytometry was performed with FACS CantoII (BD) and cell cycle data analysis was performed with FlowJo software.

### Dox, CPT and kinase inhibitor treatment

For immunofluorescence staining analysis, 1uM Dox or 20 uM CPT were applied on passage-matched normal and HGPS fibroblasts (G0/G1 synchronized or unsynchronized) for 1h. Cells were then allowed to recover for 10min (or as indicated in time course experiments) before fixation. For western blotting analysis, 0.5 uM Dox or 50 uM CPT was applied on passage-matched normal and HGPS fibroblasts for 2h before harvest. For kinase inhibition experiments, ATM, ATR or DNAPK specific inhibitors were individually applied to passage-matched normal and HGPS fibroblasts at indicated concentrations for 24h prior to Dox treatment.

### Fibroblast transfection

Transfection was performed through electroporation as previously described [11]. Briefly, 1×10^6^ cells were transfected with 4 ug plasmids (pDsRed-C1, pDsRed-C1-LA or pDsRed-C1-PG) through Amaxa NHDF Nucleofector kit (F-09376; Lonza) on a Nucleofector 2b machine (Lonza). Cells were then either seeded in 6 well plates for western blot analysis or in chamber slides for immunofluorescent staining. Doxorubicin treatment and subsequent assays were performed at 72h after transfection.

### Methylene blue treatment

Methylene blue treatment was conducted as previously described [33]. Briefly, MB was dissolved in PBS and added to growth medium at a final concentration of 100nM. Middle passage normal and HGPS fibroblasts were cultured in MB containing medium and passaged at 95% confluency. Cells were treated with MB for 30 days before analysis.

### Immunofluorescence staining

Passage-matched normal and HGPS fibroblasts were seeded in chamber slides (BD Falcon) for immunofluorescence staining. Cells were fixed with 4% PFA/PBS for 25-30min, permeabilized with 0.5% Triton X-100 (EMD Chemicals Inc.) for 5min and then blocked with 4% BSA/TBS for 1h at room temperature. Cells were then incubated with primary antibodies for overnight at 4°C. Corresponding secondary antibodies were then applied for 1h at room temperature. Fluorescence Images were taken by Zeiss LSM 710 confocal microscope.

### BrdU labeling

For BrdU labeling, cells were pre-incubated with 10 uM BrdU (BD Biosciences) for 30min before Dox treatment. To visualize BrdU, cells were treated with 2M hydrochloric acid for 30min before blocking to expose BrdU epitope. Cells were then proceeded with standard immunofluorescence staining protocol with anti-BrdU antibody. Fluorescence images were taken by Zeiss LSM 710 confocal microscope.

### Fluorescence image analysis

Fluorescence signal intensity was quantified through the “color histogram” function of Image J software (National Institute of Health, NIH). Fluorescence intensities were subtracted with background signals and plotted. The number of DNA damage associated foci (gammaH2AX, RIF1 or 53BP1) was counted through the “find maxima” function of Image J software. The average intensity of individual foci was calculated as the ratio between total fluorescence intensity and the number of foci in each nucleus. Line profile analysis was conducted through the “Line profile” plugin of Image J software.

### Western blotting analysis

Western blotting analysis was performed as previously described [11]. Briefly, cells were directly lysed in Laemmli buffer (161–0737; Bio-Rad), containing 5% (vol/vol) beta-mercaptoethanol. Protein samples were separated by 7.5% or 10% SDS-PAGE gels and transferred onto nitrocellulose membrane (Bio-Rad). Membranes were blocked with 5% milk in TBST and then incubated with primary antibodies for overnight at 4°C. Protein band intensities were quantified with Image J software (NIH).

### Statistical analysis

Two-tailed Student t test was used to analyze the difference between normal and HGPS. A p value ≤ 0.05 was considered as significant. Statistical analysis was conducted through GraphPad Prism 5.0 software.

### Correlation analysis

Spearman correlation analysis was performed to capture the association between H3K9me3 and gammaH2AX signals and estimate the correlation coefficients. We used correlation functions in R to perform the analysis and ggplot2 package to make the plots in R. Spearman rank correlation is a statistical method, which performs linear correlation test on the ranking of two variables instead of original variables to measure their monotonic relationship.

## Results

### HGPS fibroblasts show reduced gammaH2AX responses upon Dox treatment

**Fig 1:**
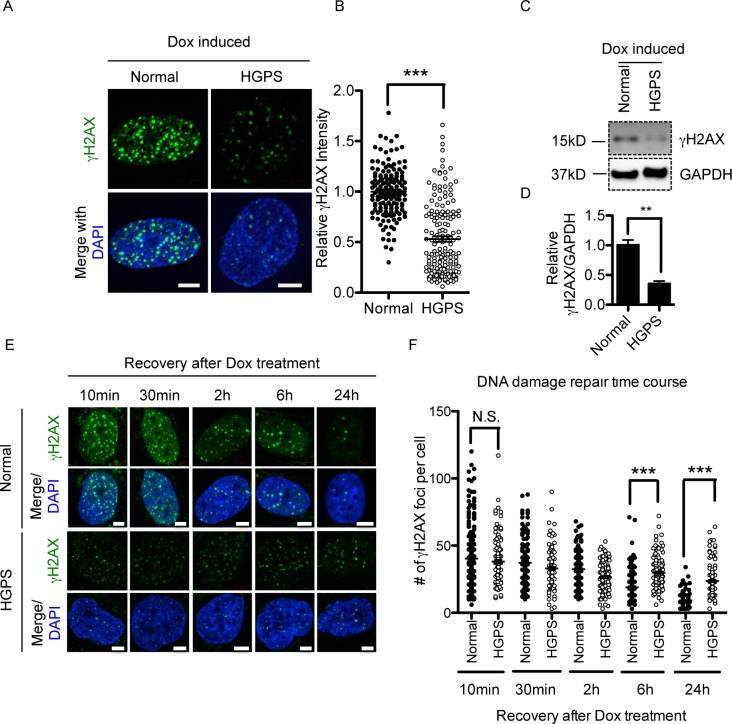
HGPS fibroblasts show reduced gammaH2AX response upon Dox treatment. (A) Representative fluorescence images of gammaH2AX foci in middle passage normal and HGPS fibroblasts after Dox treatment. Scale Bar: 5um. (B). Quantification of (A), showing relative gammaH2AX green fluorescence intensities in normal and HGPS fibroblasts after Dox treatment. More than 100 cells were randomly picked for quantification. Results were presented as mean ± SEM. ***P < 0.001. (C). Western blotting analysis with anti- gammaH2AX and anti-GAPDH antibodies on middle passage normal and HGPS fibroblasts after Dox treatment. (D). Quantification of (C), showing the relative gammaH2AX band intensity in normal and HGPS fibroblasts after Dox treatment (normalized to GAPDH). Three independent experiments were performed. Results were presented as mean ± SEM. **P < 0.01. (E).Representative fluorescence images of gammaH2AX foci in middle passage normal and HGPS fibroblasts that were allowed to recover for indicated amounts of time after Dox treatment. Scale Bar: 5um. (F). Quantification of (E), showing the number of gammaH2AX foci in each nucleus at each time point. More than 100 cells were randomly picked for quantification. Results were presented as mean ± SEM. ***P < 0.001.

To study gammaH2AX signals in HGPS, we first examined the basal number of gammaH2AX foci in middle passage (see passage definition in Materials and Methods) HGPS and normal control fibroblasts by immunofluorescence staining. As shown in S1A and S1B Fig, HGPS fibroblasts showed a slight but significant increase in the number of gammaH2AX foci than the normal control (normal: 2.5±0.3 vs. HGPS: 4.6±0.7 foci per nucleus), which is consistent with previous reports that HGPS cells accumulated more DSBs [34–36]. To further study gammaH2AX responses upon DSBs, we induced DSBs in HGPS and normal control fibroblasts by treating cells with Dox, a DNA damage-inducing agent that causes DSBs by inhibiting topoisomerase II [37]. Immunofluorescence staining and confocal microscopy analysis revealed that Dox treatment induced rapid and robust gammaH2AX responses in normal fibroblasts (Fig 1A). Notably, HGPS fibroblasts displayed significantly weaker gammaH2AX signals compared with normal cells (Fig 1A). Quantitative fluorescence analysis measuring the total fluorescence intensity of gammaH2AX further supported this reduction in HGPS (Fig 1B). Western blotting analysis showed that gammaH2AX responses were reduced in HGPS cells (Fig 1C and 1D). As the total H2AX was not significantly changed (S2 Fig), this result suggested a functional deficiency in H2AX phosphorylation.

We speculated that the reduction in gammaH2AX signals could potentially interfere with DDR in HGPS. To test this, HGPS and control fibroblasts were treated with 1uM Dox for an hour and allowed to recover for 10min, 30min, 2h, 6h, and 24h respectively. We then quantified the number of gammaH2AX foci per nuclei at each time point. As shown in Fig 1E and 1F, in normal cells, the numbers of gammaH2AX foci decreased gradually over time, indicating successful repair of the DSBs induced by Dox treatment. In contrast, while HGPS cells displayed weaker gammaH2AX signals at the beginning (10 min), these signals stayed largely unfixed even after 24h. This observation was in agreement with some previous reports describing defective DDR in HGPS [8,10,12,27].

### gammaH2AX reduction is predominantly observed in G0/G1 phase HGPS fibroblasts

**Fig 2:**
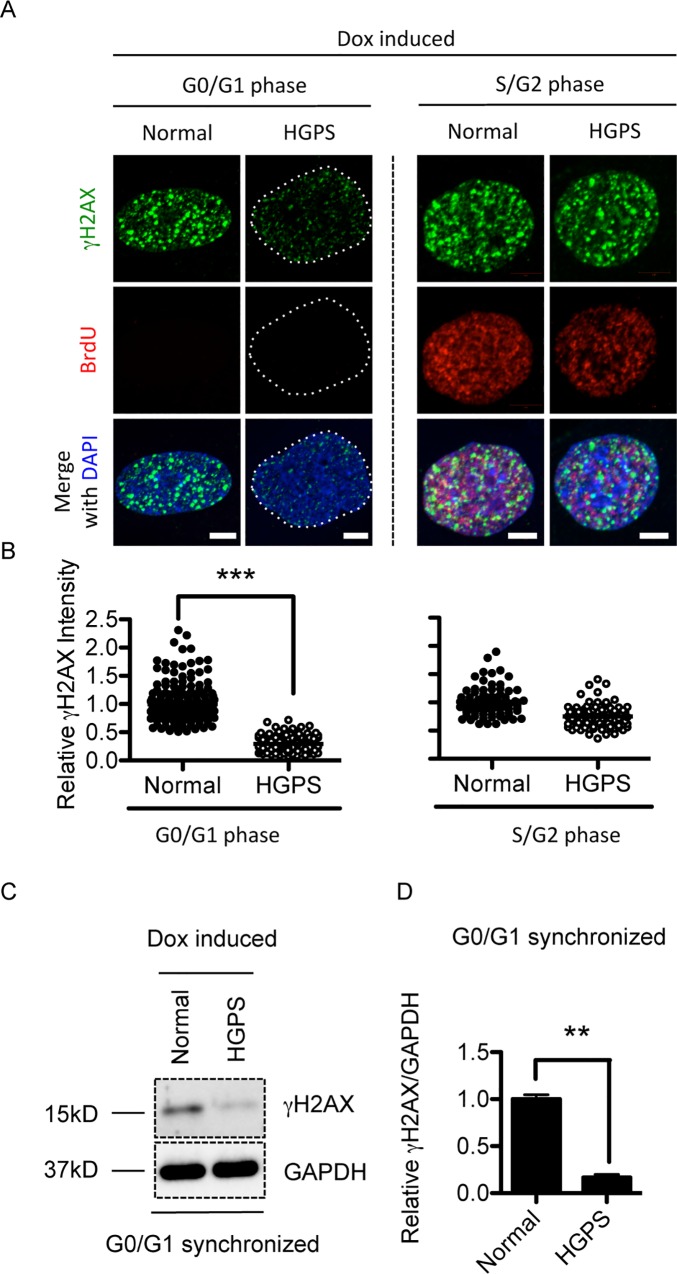
Reductions in gammaH2AX response in HGPS cells are cell cycle dependent. (A). Representative fluorescence images of gammaH2AX foci and BrdU staining in middle passage normal and HGPS fibroblasts after Dox treatment. G0/G1 and S phase were indicated by BrdU negative and positive staining respectively. Scale Bar: 5um. (B). Quantification of (A), showing relative gammaH2AX green fluorescence intensity. More than 40 cells were counted for each group. Results were presented as mean ± SEM. ***P < 0.001. (C). Western blotting analysis with anti- gammaH2AX and anti-GAPDH antibodies on serum starvation synchronized middle passage normal and HGPS fibroblasts after Dox treatment. (D). Quantification of (C), showing relative gammaH2AX band intensity (normalized to GAPDH) in serum starved normal and HGPS fibroblasts after Dox treatment. Five independent experiments were performed. Results were presented as mean ± SEM. **P < 0.01.

To investigate the gammaH2AX signal reduction phenotype in HGPS during the cell cycle, we labeled S phase cells with bromodeoxyuridine (BrdU) for 30min and then examined gammaH2AX signals in response to Dox treatment by immunofluorescence staining. We found that the BrdU-negative HGPS fibroblasts displayed a significant reduction in gammaH2AX staining (Fig 2A and 2B left panel). To our surprise, the BrdU-positive, S phase HGPS fibroblasts showed a similar level of gammaH2AX signals as normal control cells (Fig 2A and 2B right panel). Since BrdU negative staining might include both G0/G1 and G2 phase cells, to further verify the phenotype in G0/G1 phase, we synchronized normal and HGPS fibroblasts to the G0/G1 phase using serum starvation (S3 Fig). Western blotting study revealed that gammaH2AX was significantly weaker in these G0/G1 synchronized HGPS fibroblasts, compared to that in the normal control cells (Fig 2C and 2D). Taken together, these data suggest that the reduction in gammaH2AX responses mainly takes place during the G0/G1 phase in HGPS cells.

### The amplification of gammaH2AX signal is reduced in G0/G1 phase HGPS cells

**Fig 3:**
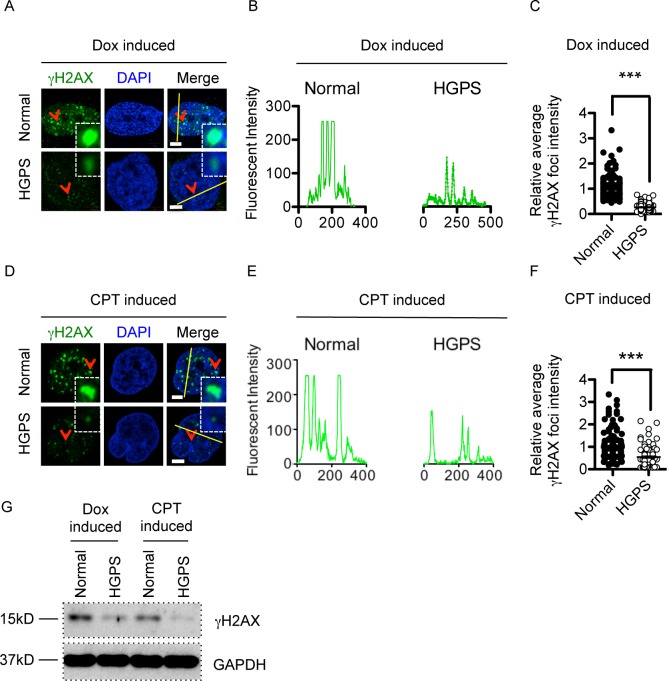
The amplification of gammaH2AX signal is reduced in G0/G1 phase HGPS cells after Dox and CPT treatment. (A). Representative fluorescence images of gammaH2AX foci in serum starvation synchronized middle passage normal and HGPS fibroblasts after Dox treatment. Arrow points to the single gammaH2AX foci in the enlarged square. Scale Bar: 5um. (B). Line profile analysis of (A), showing the reduced gammaH2AX foci fluorescence strength in HGPS fibroblasts after Dox treatment. Green fluorescence intensity (y axis) was plotted against distance (x axis) along the yellow line in (A). (C). gammaH2AX foci intensity analysis of (A). Each dot represents the average fluorescence intensity of individual gammaH2AX foci in a single nucleus. More than 100 cells were randomly picked for quantification. Results were presented as mean ± SEM. ***P < 0.001. (D). Representative fluorescence images of gammaH2AX foci in serum starvation synchronized middle passage normal and HGPS fibroblasts after CPT treatment. Arrow points to the single gammaH2AX foci in the enlarged square. Scale Bar: 5um. (E). Line profile analysis of (D), showing the reduced gammaH2AX foci fluorescence strength in HGPS fibroblasts after CPT treatment. Green fluorescence intensity (y axis) was plotted against distance (x axis) along the yellow line in (D). (F). gammaH2AX foci intensity analysis of (D). Each dot represents the average fluorescence intensity of individual gammaH2AX foci in a single nucleus. More than 100 cells were randomly picked for quantification. Results were presented as mean ± SEM. ***P < 0.001. (G). Western blotting analysis with anti- gammaH2AX and anti-GAPDH antibodies on serum starvation synchronized middle passage normal and HGPS fibroblasts after Dox or CPT treatment.

The reduction in gammaH2AX signaling could be caused by either fewer number of gammaH2AX foci or reduced intensities of individual foci. To distinguish between these two possibilities, we synchronized normal and HGPS fibroblasts to G0/G1 phase and quantified the number and the fluorescence intensity of gammaH2AX foci after Dox treatment using Image J software. Our analysis showed that HGPS and normal control fibroblasts had comparable gammaH2AX foci counts (normal: 40±1.6 vs. HGPS: 38±1.2 foci per nucleus), suggesting that similar numbers of DSBs were induced by Dox treatment in HGPS and normal cells. The result further implicated that the initial phosphorylation of H2AX at DSBs was unaffected in HGPS. On the other hand, the gammaH2AX signal amplification was disturbed in HGPS as reflected by reduced size and fluorescence intensity of individual foci (Fig 3A). Line-profile analysis and quantification of the average gammaH2AX foci intensity confirmed this observation (Fig 3B and 3C). To rule out the possibility that this phenotype was only specific to Dox-induced DSBs, we treated synchronized G0/G1 phase HGPS and normal control fibroblasts with CPT, a distinct DSB inducing agent which created DSBs by inhibiting topoisomerase I. Upon CPT treatment, HGPS and normal cells showed comparable gammaH2AX foci counts (normal: 18±0.6 vs. HGPS: 21±0.9). Consistent with Dox, CPT treatment induced significantly weaker gammaH2AX foci in HGPS G0/G1 phase fibroblasts (Fig 3D–3F). These reductions upon either Dox or CPT treatment were further validated by Western blotting analysis (Fig 3G). Together, these experiments suggested that it was not the initiation, but the amplification of gammaH2AX signaling upon DSBs that was affected in G0/G1 phase HGPS cells.

### Weakened gammaH2AX leads to a delayed recruitment of non-homologous end joining (NHEJ) factors in G0/G1 HGPS cells

**Fig 4:**
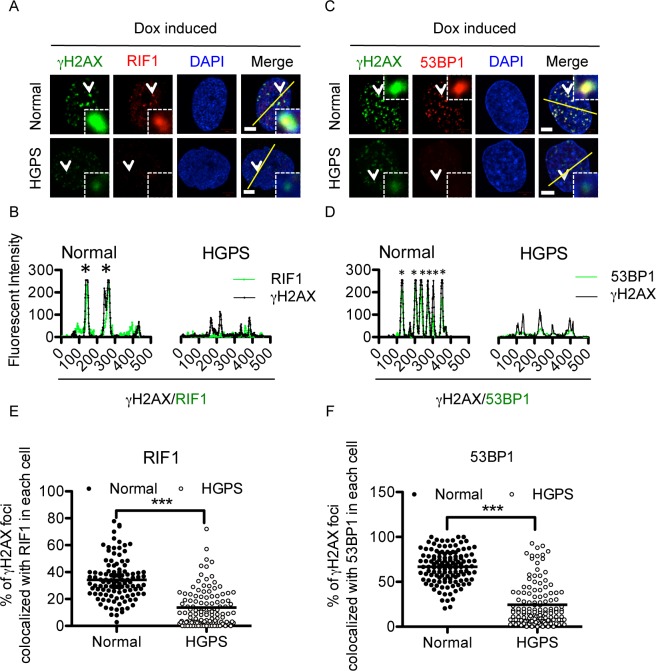
Weakened gammaH2AX leads to a delayed recruitment of non-homologous end joining (NHEJ) factors in G0/G1 HGPS cells. (A). Representative fluorescence images of gammaH2AX foci and RIF1 foci in serum starvation synchronized middle passage normal and HGPS fibroblasts after Dox treatment. Arrow points to the single gammaH2AX or RIF1 foci in the enlarged square. Scale Bar: 5um. (B). Line profile analysis of (A), showing the reduced recruitment of RIF1 to gammaH2AX foci in HGPS fibroblasts. Green (gammaH2AX) and red (RIF1) fluorescence intensities (y axis) were plotted against distance (x axis) along the yellow line in (A). Stars indicated strong co-localization of RIF1 and gammaH2AX. (C). Representative fluorescence images of gammaH2AX foci and 53BP1 foci in serum starvation synchronized middle passage normal and HGPS fibroblasts after Dox treatment. Arrow points to the single gammaH2AX or 53BP1 foci in the enlarged square. Scale Bar: 5um. (D). Line profile analysis of (C), showing the reduced recruitment of 53BP1 to gammaH2AX foci in HGPS fibroblasts. Green (gammaH2AX) and red (53BP1) fluorescence intensities (y axis) were plotted against distance (x axis) along the yellow line in (C). Stars indicated strong co-localization of 53BP1 and gammaH2AX. (E). Quantification of (A), showing the percentage of gammaH2AX foci co-localized with RIF1 foci in each nuclei in normal and HGPS fibroblasts after Dox treatment. More than 100 cells were randomly picked for quantification. Results were presented as mean ± SEM. ***P < 0.001. (F). Quantification of (C), showing the percentage of gammaH2AX foci co-localized with 53BP1 foci in each nucleus in normal and HGPS fibroblasts after Dox treatment. More than 100 cells were randomly picked for quantification. Results were presented as mean ± SEM. ***P < 0.001.

Non-homologous end joining (NHEJ) is the predominant DSB repair pathway in G0/G1 phase [11,15,38–40]. To investigate the effect of the impaired gammaH2AX signal amplification on NHEJ pathway, we examined the recruitment dynamics of RIF1, an NHEJ specific regulator, in G0/G1 phase HGPS fibroblasts [41–43]. As shown in S4A and S4B Fig, normal fibroblasts displayed robust RIF1 recruitment as early as 10min after Dox treatment while the recruitment of RIF1 did not start in HGPS cells until 2 hours later, indicating a significant delay of NHEJ response in G0/G1 phase HGPS cells. Co-localization analysis further indicated that in normal cells, RIF1 was efficiently loaded onto DSBs and co-localized with strong gammaH2AX foci (Fig 4A). Line-profile analysis confirmed this co-localization (Fig 4B). In contrast, HGPS fibroblasts didn’t show an apparent recruitment of RIF1 to DSBs, despite the presence of weak gammaH2AX foci (Fig 4A and 4B). Notably, RIF1 protein level was unchanged in HGPS fibroblasts (S4C Fig). Therefore, this delay was a functional deficiency instead of a decrease in protein amount. As a control, we also examined S phase cells and found no noticeable difference in RIF1 recruitment between normal control and HGPS cells (S4D and S4E Fig), supporting that the gammaH2AX-mediated DDR was mainly affected in G0/G1 phase HGPS cells.

Similar to RIF1, 53BP1, an important NHEJ player upstream of RIF1 [41–43], was also found to be inefficiently recruited to gammaH2AX foci in HGPS G0/G1 phase cells (Fig 4C and 4D). This observation was in agreement with a previous report that 53BP1 recruitment was disrupted in H2AX deficient mouse embryonic stem cells [44]. Importantly, 53BP1 protein level was comparable between normal and HGPS cells, suggesting that its deficiency was due to functional defects instead of reduced protein levels (S4F Fig). Quantification indicated that at the same level of DNA damage, higher percentages of gammaH2AX foci were co-localized with RIF1 or 53BP1 in normal cells compared to those in HGPS fibroblasts (Fig 4E and 4F). Collectively, these data suggest that compromised amplification of gammaH2AX signals upon DSBs affect the recruitment of NHEJ factors and delay DDR in G0/G1 phase HGPS fibroblasts.

### gammaH2AX signal reduction is associated with defective ATM activation in HGPS

**Fig 5:**
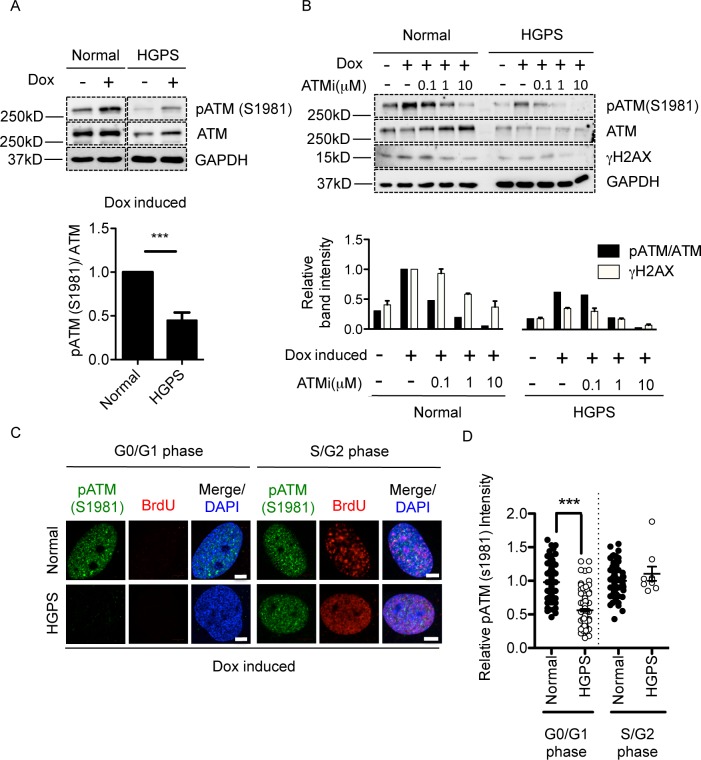
gammaH2AX signal reduction was caused by defective ATM activation in HGPS. (A). (upper): Western blotting analysis with anti-pATM(S1981), anti-ATM and anti-GAPDH antibodies on serum starvation synchronized middle passage normal and HGPS fibroblasts with or without Dox treatment. ATM activation was indicated by phosphorylated ATM(S1981). (lower): Quantification of relative pATM(S1981) band intensity (normalized to total ATM) in normal and HGPS fibroblasts after Dox treatment. Five independent experiments were performed. Results were presented as mean ± SEM. ***P < 0.001. (B). (upper): Western blotting analysis with anti-pATM(S1981), anti-ATM, anti- gammaH2AX and anti-GAPDH antibodies on serum starvation synchronized middle passage normal and HGPS fibroblasts. Cells were pre-incubated with ATM inhibitor at indicated concentrations for 24h prior to Dox treatment. (lower): Quantification of relative pATM(S1981) band intensity (normalized to total ATM) and relative gammaH2AX band intensity (normalized to GAPDH). (C). Representative fluorescence images of pATM (S1981) and BrdU staining in middle passage normal and HGPS fibroblasts after Dox treatment. G0/G1 and S phase were indicated by BrdU negative and positive staining respectively. Scale Bar: 5um. (D). Quantification of (C), showing relative pATM(S1981) green fluorescence intensity. More than 100 cells were randomly picked in each group for quantification except for S phase HGPS fibroblasts because very few S phase cells were found in that population. Results were presented as mean ± SEM. ***P < 0.001.

Three types of kinases (ATM, ATR, and DNAPK) have been previously reported to phosphorylate H2AX upon DSBs [13,18–20]. To determine which kinase mediates the phosphorylation of H2AX upon Dox treatment in G0/G1 cells, Western blotting analysis was performed to examine the activation of these three kinases in G0/G1 synchronized normal and HGPS fibroblasts. Upon Dox treatment, ATM was activated in normal cells as shown by an increased amount of phosphorylated ATM (pATM S1981) (Fig 5A) [45]. In contrast, pATM was significantly weaker in HGPS fibroblasts after Dox treatment (Fig 5A). Dosage-dependent Dox treatment further confirmed this difference between normal and HGPS fibroblasts (S5 Fig). These results were in agreement with a previous report that Zmpste24 deficient mouse embryonic fibroblasts exhibited lower ATM activation upon ionized irradiation [27]. Interestingly, the level of total ATM seemed to be also reduced in HGPS cells (Fig 5A and 5B, upper panel). This potential down-regulation of ATM protein amount could potentially contribute to the reduced pATM. However, the ratio between pATM and total ATM was still significantly lower in HGPS cells, implying additional mechanisms to impede HGPS ATM activation upon Dox treatment (Fig 5A and 5B, lower panel). Similarly, we also measured the activation of ATR and DNAPK using CHK1 phosphorylation (pCHK1, S345) and DNAPK autophosphorylation (pDNAPK, T2609), respectively. In contrast to ATM, ATR and DNAPK failed to display noticeable activation upon Dox treatment, even in the normal control cells (S6A and S6B Fig), suggesting that ATR and DNAPK may be irresponsive to Dox induced DNA damage.

It is possible that the access of the pATM or ATM antibodies, to their epitopes could be affected by nearby HGPS-specific posttranslational modifications. Thus, to directly assay whether the amplification of H2AX phosphorylation was dependent on ATM activation, we examined gammaH2AX responses in the presence of an ATM-specific inhibitor (KU-55933). As expected, incubation with the ATM inhibitor prior to Dox treatment led to a significant reduction in gammaH2AX signals in both normal and HGPS fibroblasts (Fig 5B). Quantification showed gradual reductions of both pATM and gammaH2AX with increasing amounts of the ATM inhibitor, suggesting that ATM activity was indispensable for gammaH2AX responses upon Dox treatment (Fig 5B, lower panel). Since gammaH2AX signals were crucial for 53BP1 recruitment at DSBs, we wondered whether ATM inhibitor could directly impair 53BP1 recruitment. Notably, normal fibroblasts treated with ATM inhibitor showed a significantly reduced recruitment of 53BP1 to DSBs compared to non-treated control (non-treated: 84% vs. ATM inhibitor: 27%, showing more than five 53BP1 foci per nucleus) (S7A–S7C Fig). These results were in agreement with previous reports that ATM activity was crucial for gammaH2AX signal amplification and 53BP1 recruitment [37,46]. In contrast to ATM inhibitor, ATR inhibitor showed no apparent effects on gammaH2AX signals while DNAPK inhibitor was only able to slightly reduce gammaH2AX responses at the highest concentration of 10 μM (S6C and S6D Fig), further suggesting that ATM was the major governor of gammaH2AX responses upon Dox treatment.

Furthermore, to understand why the gammaH2AX reduction in HGPS was G0/G1 phase specific, we checked the level of pATM in G0/G1 and S phase HGPS fibroblasts upon Dox treatment. As shown in Fig 5C and 5D, the immunofluorescence staining revealed that ATM activation was significantly inhibited in G0/G1 phase but not in S phase HGPS fibroblasts (Fig 5C and 5D), which was in high accordance with the observed differential levels of gammaH2AX in G0/G1 and S phases HGPS cells (Fig 2A and 2B). Together, these results indicate that ATM activation is responsible for the amplification of gammaH2AX signals upon Dox treatment and that in G0/G1 phase HGPS cells, proper ATM activation was impaired.

### Loss of H3K9me3 correlates with impair ATM activation in HGPS

It has been recently shown that histone H3 trimethylated on lysine 9 (H3K9me3) was crucial for ATM activation [29,30], in particular, the removal of H3K9me3 by depletion of Suppressor of Variegation 3–9 Homolog 1 (SUV39h1, the methyltransferase that methylates H3K9) completely blocked ATM activation upon DSBs [29]. It is worth mentioning that the trimethyl mark of H3K9 is diluted during replication in the S phase, and the newly synthesized histones become fully methylated only in the next G1 phase [47]. In HGPS cells, a dramatic loss of H3K9me3 has frequently been observed [6,33,48,49]. Based on this information, we hypothesized that loss of H3K9me3 in HGPS cells caused the consequent blockage of ATM activation, resulting in insufficient gammaH2AX signals at the DSBs and delayed DDR.

**Fig 6:**
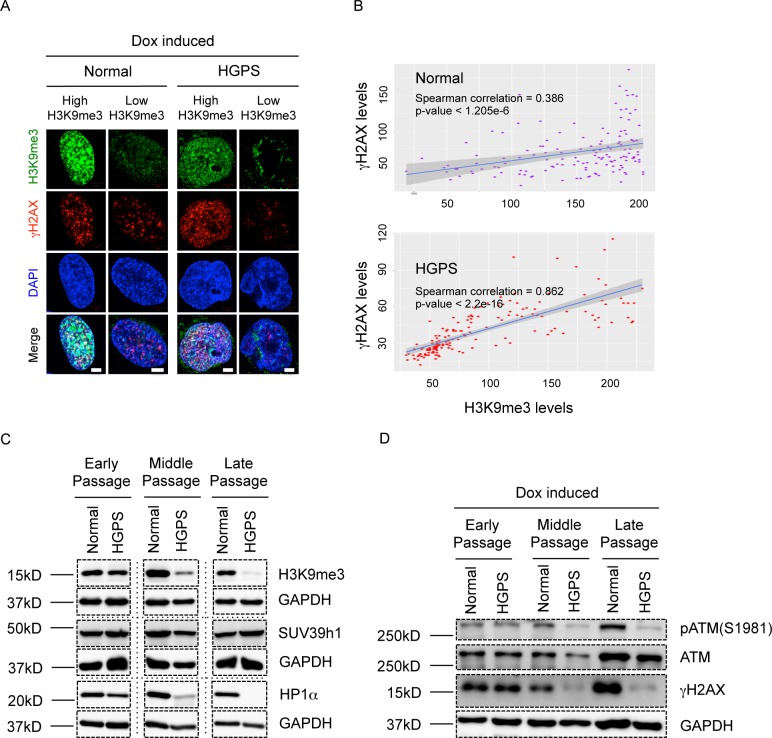
Loss of H3K9me3 correlates with impaired ATM activation in HGPS. (A). Representative fluorescence images of gammaH2AX and H3K9me3 in middle passage normal and HGPS fibroblasts after Dox treatment. “Low H3K9me3” is defined as any H3K9me3 fluorescence intensity that falls below the lower 5% of the normal population. Scale Bar: 5um. (B). Quantification of (A), showing the Spearman correlations between H3K9me3 green fluorescence intensity and gammaH2AX red fluorescence intensity in normal and HGPS fibroblasts. More than 100 cells were randomly picked for quantification in each group. (C). Western blotting analysis with anti-H3K9me3, anti-SUV39h1, anti-HP1alpha and anti-GAPHD antibodies on normal and HGPS fibroblasts at early, middle and late passages respectively. (D). Western blotting analysis with anti-pATM(S1981), anti-ATM, anti- gammaH2AX and anti-GAPDH antibodies on serum starvation synchronized normal and HGPS fibroblasts at early, middle and late passages after Dox treatment.

To test this hypothesis, we first examined the correlation between H3K9me3 and gammaH2AX signals in normal and HGPS cells. As shown in Fig 6A, we found that after Dox treatment cells with robust gammaH2AX responses almost always displayed decent H3K9me3 staining, and weak gammaH2AX staining was often observed in cells with decreased H3K9me3. Spearman correlation analysis based on the fluorescence intensities of both indicated a significant positive correlation between H3K9me3 and gammaH2AX in both normal and HGPS fibroblasts (Fig 6B).

It has been previously established that premature senescence, chromatin disorganization and epigenetic abnormalities (loss of H3K9me3 and H3K27me3) worsen in HGPS cells as progerin accumulated with increased cellular passages [6,48,50,51]. We asked whether ATM activation and H2AX phosphorylation also followed this pattern. To test this, we synchronized early, middle and late passage HGPS and normal control fibroblasts to G0/G1 phase through serum starvation, treated them with Dox and examined their levels of pATM and gammaH2AX. In agreement with the previous report, H3K9me3 level was high and similar to the controls at early passage but became gradually reduced at middle and late passages (Fig 6C) [48]. Moreover, both pATM and gammaH2AX signals displayed a similar pattern of deterioration in HGPS cells with increased cellular passages (Fig 6D). Together, these results demonstrated a strong correlation between the H3K9me3 levels and the ATM activation and H2AX responses in HGPS cells, suggesting that loss of H3K9me3 could potentially impair ATM activation upon DSBs in G0/G1 phase HGPS cells.

Significantly, the reductions in H3K9me3 and DDR were accompanied by a passage dependent accumulation of progerin in HGPS, supporting an inhibitory role of progerin in this pathway (S8 Fig). In addition, normal cells ectopically expressing progerin failed to display a robust ATM activation upon Dox treatment, whereas cells over expressing wild type control lamin A showed normal ATM activation (S9A and S9B Fig), indicating that progerin plays a direct inhibitory role on DDR.

To provide some insights into the down-regulation of H3K9me3 in HGPS cells, we first examined SUV39h1. Our analysis indicated that the expression of SUV39h1 was similar in normal and HGPS cells, even at late cellular passages (Fig 6C), suggesting that the reduction in H3K9me3 might not be caused by a decrease in its methylation. We next measured heterochromatin protein 1αeter (HP1αHP1r), a heterochromatin binding protein that had been shown to play a crucial role in maintaining H3K9me3 [52]. Interestingly, HP1alpha displayed a passage dependent reduction in HGPS fibroblasts, suggesting that the down-regulation of HP1alpha might be responsible for H3K9me3 loss in HGPS (Fig 6C). HP1aalpha has been shown to facilitate the recruitment of SUV39h1 in response to DNA damage, which further mediates a transient H3K9me3 enrichment and initiates the DDR signals at local DSBs [30]. Thus, we further tested whether HP1alpha reduction would impact SUV39h1 recruitment in HGPS cells. As shown in S10A and S10B Fig, normal fibroblasts displayed a strong SUV39h1 foci formation after Dox treatment, whereas HGPS cells failed to show as many SUV39h1 foci, implying that DSB induced SUV39h1 recruitment was disrupted in HGPS cells.

### Methylene blue rescues H3K9me3 loss and DDR defects in HGPS

Methylene blue (MB) treatment has been shown to maintain progerin level but increase its solubility by releasing it from the inner nuclear membranes in HGPS cells [33]. Long-term treatment (~6 weeks) with MB was able to rescue HP1alpha reduction and heterochromatin loss in HGPS fibroblasts [33]. We, therefore, speculated that MB treatment might restore H3K9me3 level and rescue the downstream defects of H3K9me3 loss, including the weakened ATM and H2AX phosphorylation and the delayed DDR in HGPS.

**Fig 7:**
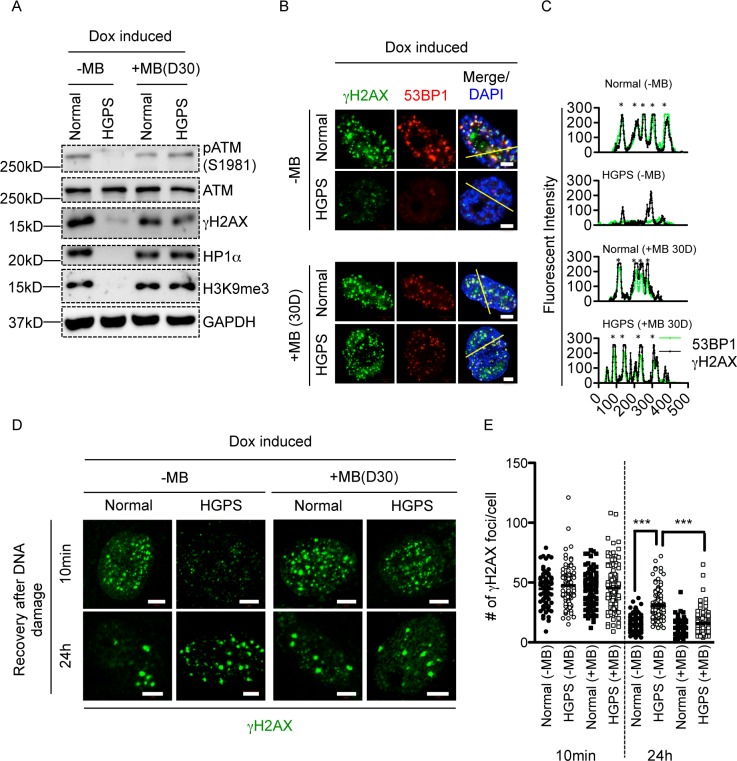
Methylene blue restores H3K9me3 and rescues DDR defects in HGPS. (A). Western blotting analysis with anti-pATM(S1981), anti-ATM, anti- gammaH2AX, anti-HP1alpha, anti-H3K9me3 and anti-GAPDH antibodies on late passage normal and HGPS fibroblasts treated with or without MB after 30 days. Cells were treated with Dox to induce DSBs before analysis. Two independent western blotting analysis were performed. (B). Representative fluorescence images of gammaH2AX foci and 53BP1 foci in normal and HGPS fibroblasts treated with or without MB for 30 days. Cells were treated with Dox to induce DSBs before analysis. Scale Bar: 5um. (C). Line profile analysis of (B), showing the robust recruitment of 53BP1 to gammaH2AX in MB treated HGPS fibroblasts. Green (gammaH2AX) and red (53BP1) fluorescence intensities (y axis) were plotted against distance (x axis) along the yellow line in (B). Stars indicated strong co-localization of 53BP1 to gammaH2AX. (D). Representative fluorescence images of gammaH2AX foci in late passage normal and HGPS fibroblasts treated with or without MB after 30 days. Cells were then treated with Dox to induce DSBs and allowed to recover for indicated amounts of time before analysis. Scale Bar: 5um. (E). Quantification of (D), showing the number of gammaH2AX foci in each nucleus at each time point. More than 100 cells were randomly picked for quantification in each group. Results were presented as mean ± SEM. ***P < 0.001.

Normal and HGPS fibroblasts were treated with 100nM MB for 30 days and then synchronized to G0/G1 phase through serum starvation and tested for ATM and gammaH2AX response upon Dox treatment. As expected, non-treated G0/G1 phase HGPS fibroblasts displayed reduced HP1alpha compared to normal control, whereas HP1alpha level was drastically increased in MB treated cells over the 30-day treatment (Fig 7A). Moreover, H3K9me3 loss was rescued in G0/G1 phase HGPS fibroblasts after MB treatment, further supporting the beneficial effects of MB on heterochromatin architecture in HGPS cells (Fig 7A) [33]. Importantly, ATM and gammaH2AX responses upon Dox induction were greatly restored in MB treated HGPS fibroblasts, whereas MB itself didn’t elicit any ATM activation. (Fig 7A, S11A and S11B Fig). Moreover, cells with increased gammaH2AX responses also displayed higher amounts of H3K9me3 in MB treated HGPS cells (S12A and S12B Fig), suggesting that MB may benefit DDR through improving H3K9me3. To test whether the up-regulation of gammaH2AX responses by MB was sufficient to overcome NHEJ deficiency in HGPS, we measured 53BP1 recruitment in MB treated HGPS fibroblasts. As shown in Fig 7B, at 10min after Dox treatment, 53BP1 was more efficiently recruited to gammaH2AX foci in MB treated than untreated HGPS fibroblast, suggesting a beneficial effect of MB on the impaired NHEJ in HGPS fibroblasts. Line profile analysis also confirmed the co-localization between 53BP1 and gammaH2AX in MB treated HGPS fibroblasts (Fig 7C). Next, we investigated whether DDR efficiency was restored in HGPS after MB treatment by examining gammaH2AX foci turn over in MB treated normal and HGPS fibroblasts. As shown in Fig 7D and 7E, within 24h after Dox treatment, gammaH2AX foci count dropped drastically from 44±1.7/nucleus to 16±0.7/nucleus in MB treated HGPS fibroblasts. In contrast, non-treated HGPS fibroblasts exhibited a significant delay in DNA damage repair as shown by retaining a substantial amount of gammaH2AX foci (31±1.1 foci/nucleus) after 24hours recovery (Fig 7E). In comparison, normal fibroblasts showed active DDR with/without MB treatment (24h after Dox treatment, -MB: 16±0.5 and +MB: 12±0.5 foci/nucleus) (Fig 7E). Taken together, these data provided additional support to our hypothesis that increasing H3K9me3 could potentially promote ATM activation and gammaH2AX amplification and rescue DDR deficiency in HGPS cells.

## Discussion

### Attenuated gammaH2AX signals in response to DSBs in HGPS cells

It has been previously reported that ATM activation was impaired in Zmpste24^-/-^ MEFs upon ionized irradiation [27]. This deficiency was proposed to impede chromatin remodeling upon DSBs in HGPS cells. As the most conspicuous histone modification at DSBs, histone variant H2AX can be directly phosphorylated by ATM to form gammaH2AX. This modification plays a critical function in DDR, including recruitment of essential DDR proteins to the DSB site to repair. [13,15,17]. In this study, we reported that H2AX phosphorylation was significantly reduced in G0/G1 phase HGPS fibroblasts upon Dox treatment (Figs 1 and 2). Notably, reduced gammaH2AX responses can be directly caused by upstream ATM inhibition and are associated with downstream delayed 53BP1 and RIF1 recruitments in HGPS fibroblasts (Figs 4 and 5). This finding serves to bridge the gap between the previously known ATM deficiency and the delayed DDR repair factor recruitment in progeria.

Interestingly, although we observed DDR deficiencies in HGPS cells, we didn’t observe increased basal level DNA damage in late passage HGPS cells. We reasoned that this could be due to the selective effect of *in vitro* cell culture, as cells with severe DNA damage may not survive the serial passaging process. In addition, according to our model, HGPS cells bear reduced gammaH2AX signaling with DSBs. Therefore, a low level of gammaH2AX at basis doesn’t necessarily reflect a low level of DNA damages at late passage.

### Reductions in gammaH2AX and ATM activity in HGPS cells are cell cycle dependent

We distinguished replicating cells from the rest by BrdU labeling and uncovered a previously unknown cell cycle dependent reduction of ATM and gammaH2AX responses in HGPS. Unlike G0/G1 phase cells which bear reduced ATM and gammaH2AX responses, S phase HGPS fibroblasts displayed normal gammaH2AX signals and ATM activation upon Dox induction (Fig 2A and 2B, Fig 5C and 5D). An intriguing question is why ATM activation is not blocked in the S phase HGPS cells? One possibility is that the chromatin compaction and organization are different between G0/G1 and S phase cells. For instance, during the process of DNA replication, chromatin opens up and renders a more flexible structure, which has been shown to favor the recruitment of DDR pathway proteins [53]. Supporting this idea, the previous study has demonstrated that “opening up” chromatin by depleting histone H1 or treating cells with histone deacetylase (HDAC) inhibitor TSA was able to significantly enhance gammaH2AX signals upon DSBs [54]. Apart from gammaH2AX, alterations in chromatin structure also directly impact ATM activity. “Opening up” chromatin through TSA treatment has been shown to activate ATM either with or without the presence of DSBs [45,55]. Thus, it is likely that the chromatin structure remodeling during S phase might facilitate ATM activation and phosphorylate H2AX upon DSBs, alleviating the inhibition in HGPS.

The cell cycle dependent reduction of ATM and gammaH2AX responses implied a possibility of distinct molecular mechanisms underlying HGPS G0/G1 or S phase DDR mis-regulations. For instance, in this study, we observed that NHEJ was significantly delayed in G0/G1 phase but unaffected in S phase HGPS fibroblasts (S4 Fig). Moreover, in our previous study on induced pluripotent stem cell (iPSC) differentiated HGPS SMCs, we revealed an over-activation of NHEJ during S phase, leading to chromosome aberrations and mitotic catastrophe [11]. These results suggested a differential regulation of NHEJ DSB repair pathway between G0/G1 and S phase HGPS cells. Reduced ATM/ gammaH2AX signals might impair NHEJ during G0/G1 phase while robust ATM/ gammaH2AX signals promote NHEJ during S phase, providing a plausible mechanism underlying the mis-regulations of NHEJ in HGPS cells at different cell cycle phases.

### Loss of H3K9me3 correlates with DDR deficiencies in HGPS cells

Abnormal histone modification has long been proposed to contribute to DDR deficiency in HGPS. One previous study from Liu et al. claimed that H3K9me3 accumulation established a chromosomal barrier preventing DDR players from being recruited [28]. Interestingly, in our system, we observed a passage dependent reduction of H3K9me3 and found a significant correlation between H3K9me3 loss and the reduced ATM and gammaH2AX responses in HGPS cells (Fig 6). This was a novel correlation in progeria and was in agreement with previous studies that H3K9me3 was crucial for ATM activation upon DSBs [29,30].

There is currently no general mechanism to explain the depletion of H3K9me3 in HGPS cells. Interestingly, this phenotype was accompanied by a down-regulation of HP1alpha, one of the most prominent binding partners of H3K9me3 (Fig 6) [48,56]. Chromatin bound HP1alpha further recruits SUV39h1, which methylates nearby H3K9, forming a positive feedback loop to regulate H3K9me3 level [56]. Depletion of HP1alpha leads to H3K9me3 loss and cellular senescence, supporting a critical role of HP1alpha in H3K9me3 maintenance [52]. In addition, upon DSBs, HP1alpha forms a complex with SUV39h1 and Kap1 and regulates local methylation on H3K9, creating a transient H3K9me3 peak around the DNA lesions to facilitate ATM activation and DDR [30]. This process was disrupted by over expression of HP1alpha dominant negative mutant [30]. Significantly, we observed a reduced recruitment of SUV39h1 in HGPS cells upon DSBs, which could potentially impair local H3K9me3 enrichment and DDR signaling (S10 Fig). The above information highlights a potential destructive role of HP1alpha loss in H3K9me3 maintenance and DDR in HGPS.

Interestingly, Booth et al. have shown that loss of higher order chromatin structure resulted in increased chromatin compliance in HGPS cells as well as in cells ectopically expressing progerin [57]. Since chromatin dynamics is associated with chromatin translocation frequency [58,59]. Those findings may suggest that H3K9me3 loss induced chromatin decondensation can also contribute to HGPS genome instability by perturbing chromatin dynamics.

### The role of progerin and premature senescence in HGPS DDR deficiency

The phenotypes of H3K9me3 loss and ATM and gammaH2AX signal reductions were becoming more severe with prolonged passaging in HGPS cells. We speculated that this was due to the accumulation of progerin in HGPS cells over passages. This hypothesis was supported by previous reports that progerin over-expression directly induced H3K9me3 loss [48,49]. Moreover, we also observed a direct inhibitory role of progerin on ATM activation upon Dox treatment (S9 Fig). These pieces of evidence supported a negative correlation between progerin and ATM and gammaH2AX responses.

Aside from accumulating progerin, HGPS fibroblasts also became prematurely senescent over passages, raising the possibility that cellular senescence plays a secondary role on H3K9me3 loss and DDR deficiency in HGPS cells. In support of this idea, Scaffidi et al. have previously shown that fibroblasts from normal donors exhibited a gradual reduction of H3K9me3 with prolonged passages [60]. In addition, fibroblasts from senior individuals displayed significantly reduced H3K9me3 compared to those from young individuals, implying a role of human aging on H3K9me3 loss [52,60]. Given the role of H3K9me3 in ATM activation upon DSBs, its loss might impair ATM and gammaH2AX responses in senescent cells, suggesting a negative role of senescence on ATM activity. Interestingly, persistent activation of ATM in response to DNA damage is widely considered as the upstream signal of cellular senescence [61–63]. Based on the above chain of evidence, we would like to propose that there might a negative feedback response of ATM activation to cellular senescence. At the down-stream of DDR, both NHEJ and HR DSB repair pathways were shown to be less efficient in senescent cells, supporting that senescence undermined DNA damage repair and genome stability [64,65].

### Methylene blue restores H3K9me3 and DDR in HGPS cells

Previously, we showed that methylene blue (MB) was able to improve mitochondrial functionality, alleviate nuclear blebbing and restore heterochromatin loss in HGPS fibroblasts [33]. Interestingly, this beneficial effect seemed to require long-term treatment (4-6weeks) [33]. Mechanistically, in addition to its anti-oxidative role in mitochondria, MB was shown to increase the solubility of progerin and relocate it from the inner nuclear membrane into nucleoplasm [33]. We speculated that this process might be relatively slow, as the turnover rate of “bad” mitochondria is 8–23 days depending on cell types. Given the beneficial effects of MB on heterochromatin, we wondered whether MB was able to rescue H3K9me3 and subsequent ATM and gammaH2AX responses in HGPS. Indeed, MB almost completely restored H3K9me3 level, ATM activation, gammaH2AX signals and subsequent DNA damage repair in G0/G1 HGPS fibroblasts (Fig 7). MB possesses a wide range of beneficial effects on HGPS, potentially due to its perturbation on progerin solubility [33]. In this sense, we cannot rule out the possibility that MB improved DDR through mechanisms in addition to H3K9me3 restoration. For example, we showed that MB was able to fix the disrupted global gene expression profile in HGPS fibroblasts [33], suggesting broad downstream effects, some of which might improve the deficient DDR. Future studies are required to identify and validate these mechanisms. Based on these results, our study pointed out a novel therapeutic approach to treat the defective DDR in HGPS.

## Supporting Information

S1 Fig HGPS fibroblasts bear elevated gammaH2AX count at basal level (A) Representative fluorescence images of basal gammaH2AX foci in middle passage normal and HGPS fibroblasts. Scale Bar: 5um.
(B) Quantification of (A) showing the number of gammaH2AX foci in each nucleus in middle passage normal and HGPS fibroblasts at a basal level. More than 100 cells were randomly picked for quantification. Results were presented as mean ± SEM. **P < 0.01.
(TIF)

S2 Fig HGPS and normal control fibroblasts display equal amount of histone H2AX Western blotting analysis with anti-H2AX, anti-alpha-tubulin and anti-GAPDH antibodies on middle passage normal and HGPS fibroblasts.
(TIF)

S3 Fig Cell cycle analysis of serum starvation synchronized HGPS and normal fibroblasts Cell cycle analysis of middle passage normal and HGPS fibroblasts with or without serum starvation synchronization.
(TIF)

S4 Fig RIF1 recruitment was delayed in G0/G1 phase but not in S/G2 phase HGPS fibroblasts (A) Representative fluorescence images of RIF1 and BrdU in middle passage normal and HGPS cells. G0/G1 cells were indicated by BrdU negative staining. Scale Bar: 5um.
(B) Quantification of (A), showing the number of RIF1 foci in each nucleus at each time point. More than 50 cells were picked for each group. Results were presented as mean ± SEM. ***P < 0.001.
(C) Western blotting analysis with anti-RIF1 and anti-GAPDH antibodies in middle passage normal and HGPS fibroblasts.
(D) Representative fluorescence images of RIF1 and BrdU in middle passage normal and HGPS cells. S phase cells were indicated by BrdU positive staining. Scale Bar: 5um.
(E) Quantification of (D), showing the number of RIF1 foci in each nucleus at each time point. More than 100 cells were picked. Results were presented as mean ± SEM.
(F) Western blotting analysis with anti-53BP1 and anti-GAPDH antibodies in middle passage normal and HGPS fibroblasts.
(TIF)

S5 Fig HGPS fibroblasts displayed reduced ATM activation upon DSBs Western blotting analysis with anti-pATM(S1981), anti-ATM, anti- gammaH2AX and anti-GAPDH antibodies in middle passage normal and HGPS fibroblasts treated with indicated concentrations of Dox.
(TIF)

S6 Fig ATR and DNAPKcs were inactivated upon DSBs (A) Western blotting analysis with anti-pCHK1(S345), anti-CHK1 and anti-GAPDH antibodies on middle passage normal and HGPS fibroblasts after Dox treatment. ATR activation was indicated by phosphorylation of CHK1(S345).
(B) Western blotting analysis with anti-pDNAPK(T2609), anti-DNAPK and anti-GAPDH antibodies on middle passage normal and HGPS fibroblasts after Dox treatment. DNAPK activation was indicated by phosphorylation of DNAPK(T2609).
(C) Western blotting analysis with anti-pCHK1(S345), anti-CHK1, anti- gammaH2AX and anti-GAPDH antibodies on middle passage normal and HGPS fibroblasts pre-incubated with indicated concentrations of ATR inhibitor for 24h prior to Dox treatment.
(D) Western blotting with anti-pDNAPK (T2609), anti-DNAPK, anti- gammaH2AX and anti-GAPDH antibodies on middle passage normal and HGPS fibroblasts pre-incubated with indicated concentrations of DNAPKcs inhibitor for 24h prior to Dox treatment.
(TIF)

S7 Fig ATM inhibitor reduces 53BP1 recruitment (A) Representative fluorescence images of gammaH2AX and 53BP1 in middle passage normal fibroblasts pre-incubated with or without 10uM ATM specific inhibitor prior to Dox treatment. Scale Bar: 5um.
(B) Line profile analysis of (A), showing the reduced recruitment of 53BP1 to gammaH2AX foci in ATM inhibitor treated fibroblasts. Green (gammaH2AX) and red (53BP1) fluorescence intensities (y axis) were plotted against distance (x axis) along the yellow line in (A). Stars indicated strong co-localization of 53BP1 and gammaH2AX.
(C) Quantification of the percentage of a population that displayed more than five 53BP1 foci in control or ATM inhibitor treated fibroblasts. Results were presented as mean ± SEM. ***P < 0.001.
(TIF)

S8 Fig Progerin accumulates in HGPS fibroblasts along passages (A) Western blotting analysis with anti-Lamin A/C and anti-GAPDH antibodies on normal and HGPS fibroblasts at early, middle and late passages, showing the passage dependent accumulation of progerin in HGPS.
(B) Quantification of (A), showing the relative band intensity of progerin (normalized to GAPDH) in early, middle and late passage HGPS fibroblasts.
(TIF)

S9 Fig Progerin directly inhibits ATM activation upon DSBs. (A) Representative fluorescence images of Dox induced pATM(S1981) in late passage normal fibroblasts ectopically expressing DsRed, DsRed-lamin A (DsRed-LA) and DsRed-progerin (DsRed-PG) respectively. Scale Bar: 5um.
(B) Quantification of (A), showing the fluorescence intensities of DsRed (x axis) and pATM(S1981) (y axis) in either DsRed-LA or DsRed-PG over expressing cells, after Dox treatment. Trend line and R^2^ were calculated using linear regression function.
(C) Western blotting analysis with anti-pATM(S1981), anti-ATM, anti-lamin A/C and anti-GAPHD antibodies on late passage normal fibroblasts ectopically expressing DsRed, DsRed-lamin A (DsRed-LA) or DsRed-progerin (DsRed-PG). Cells were treated with or without Dox to induce DSBs before analysis.
(D) Quantification of (C), showing the relative band intensity of pATM over total ATM before or after Dox treatment. Three independent experiments were performed. Results were presented as mean ± SEM. *P < 0.05.
(TIF)

S10 Fig SUV39H1 foci formation is disrupted in HGPS fibroblasts upon DSBs. (A) Representative fluorescence images of Dox induced SUV39H1 foci formation in middle passage normal and HGPS fibroblasts. Scale Bar: 5um.
(B) Quantification of (A), showing the number of SUV39H1 foci in each cell after Dox treatment. More than 100cells were randomly picked for each group. Results were presented as mean ± SEM. ***P < 0.001.
(TIF)

S11 Fig Methylene blue treatment doesn’t elicit ATM activation in HGPS cells at a basal level. (A) Western blotting analysis with anti-pATM(S1981), anti-ATM and anti-GAPDH antibodies on late passage normal and HGPS fibroblasts with indicated treatments.
(B) Quantification of (A), showing that the relative band intensity of pATM(S1981) over total ATM was unchanged at basal level (without Dox induction) in HGPS cells with/out methylene blue treatment.
(TIF)

S12 Fig Methylene blue restores H3K9me3 and enhances gammaH2AX signals in HGPS cells upon DSBs. (A) Representative fluorescence images of H3K9me3 and gammaH2AX responses in late passage normal and HGPS fibroblast with or without methylene blue treatment. Scale Bar: 5um.
(B) Quantification of (A), showing the Spearman correlations between H3K9me3 green fluorescence intensity and gammaH2AX red fluorescence intensity in HGPS fibroblasts with or without methylene blue treatment. More than 100 cells were randomly picked for quantification in each group.
(TIF)
