# Supplementary figures and images for: Loss of H3K9me3 Correlates with ATM Activation and Histone H2AX Phosphorylation Deficiencies in Hutchinson-Gilford Progeria Syndrome
Source: PLoS One. 2016 Dec 1;11(12):e0167454. doi: 10.1371/journal.pone.0167454 (PMC5131972; doi:10.1371/journal.pone.0167454)

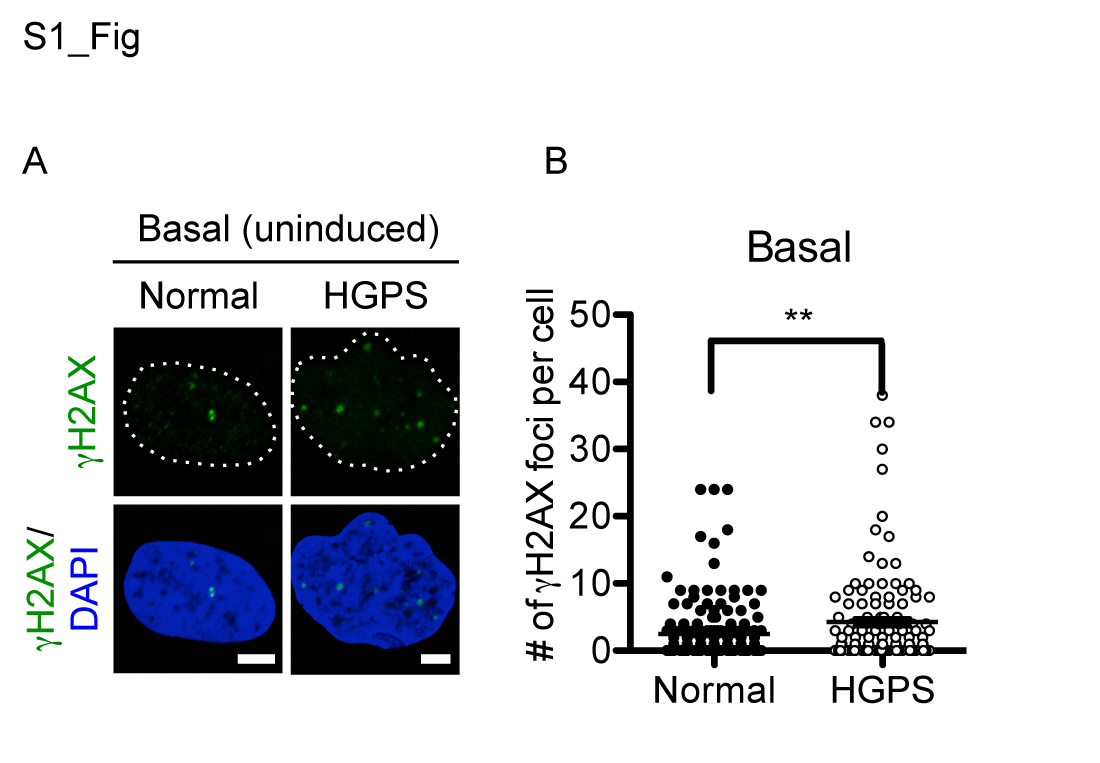

Supplement: S1 Fig — (A) Representative fluorescence images of basal gammaH2AX foci in middle passage normal and HGPS fibroblasts. Scale Bar: 5um. (B) Quantification of (A) showing the number of gammaH2AX foci in each nucleus in middle passage normal and HGPS fibroblasts at a basal level. More than 100 cells were randomly picked for quantification. Results were presented as mean ± SEM. **P < 0.01. (TIF) [file pone.0167454.s001.tif]

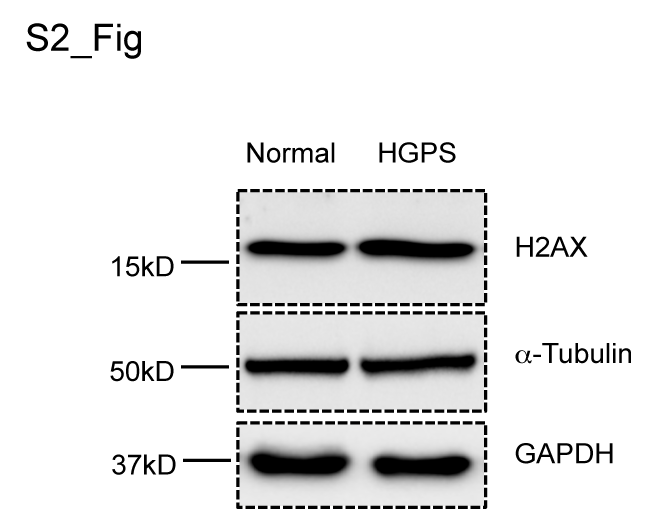

Supplement: S2 Fig — Western blotting analysis with anti-H2AX, anti-alpha-tubulin and anti-GAPDH antibodies on middle passage normal and HGPS fibroblasts. (TIF) [file pone.0167454.s002.tif]

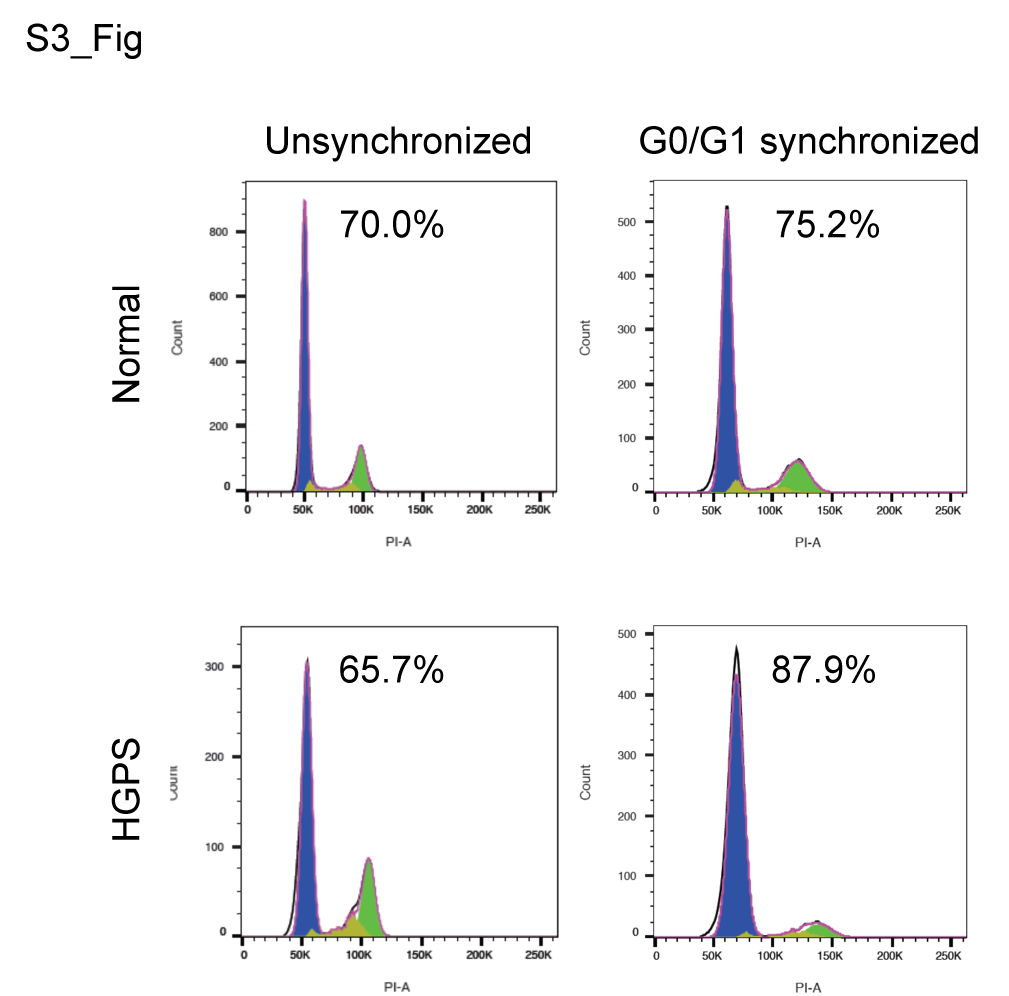

Supplement: S3 Fig — Cell cycle analysis of middle passage normal and HGPS fibroblasts with or without serum starvation synchronization. (TIF) [file pone.0167454.s003.tif]

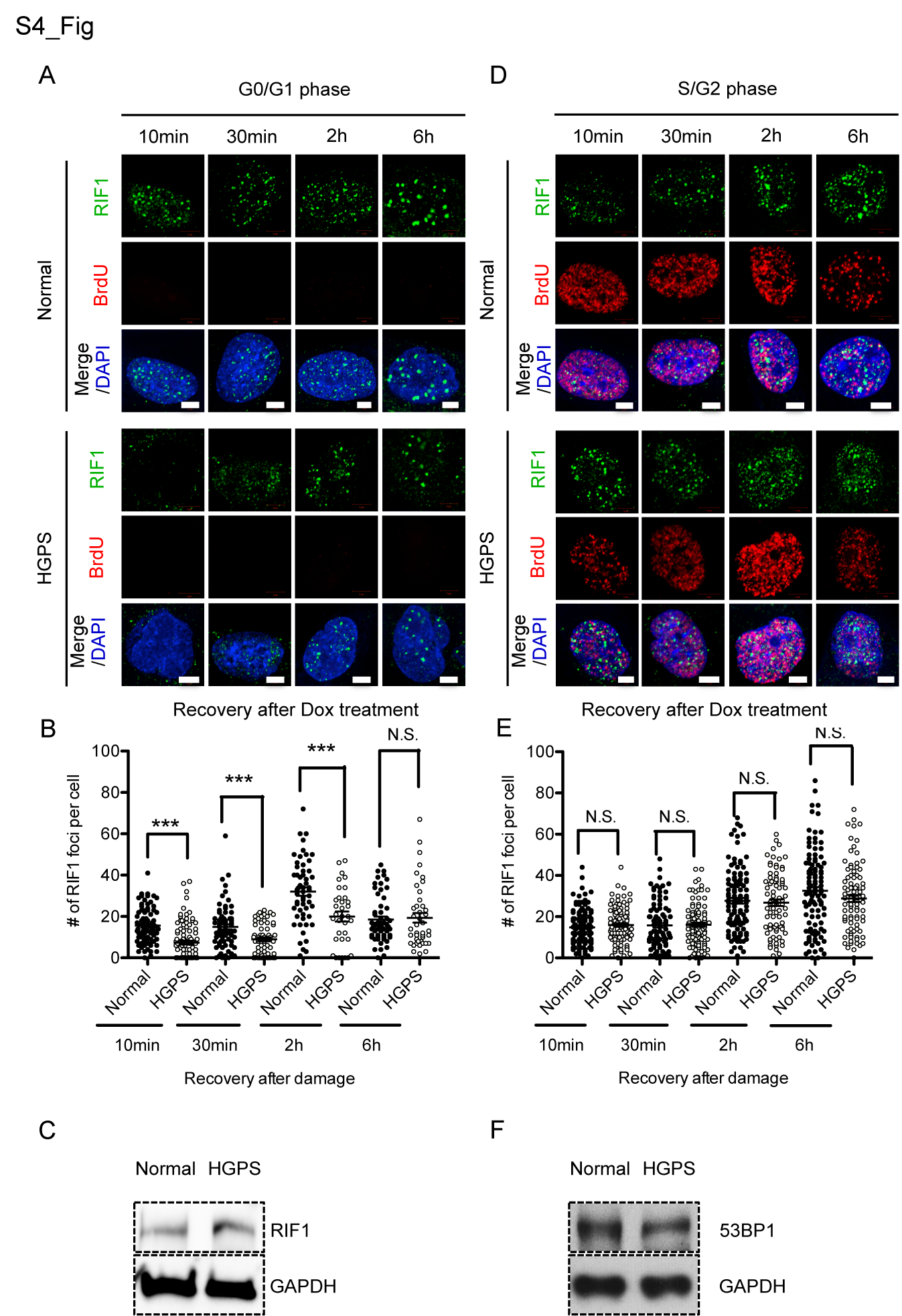

Supplement: S4 Fig — (A) Representative fluorescence images of RIF1 and BrdU in middle passage normal and HGPS cells. G0/G1 cells were indicated by BrdU negative staining. Scale Bar: 5um. (B) Quantification of (A), showing the number of RIF1 foci in each nucleus at each time point. More than 50 cells were picked for each group. Results were presented as mean ± SEM. ***P < 0.001. (C) Western blotting analysis with anti-RIF1 and anti-GAPDH antibodies in middle passage normal and HGPS fibroblasts. (D) Representative fluorescence images of RIF1 and BrdU in middle passage normal and HGPS cells. S phase cells were indicated by BrdU positive staining. Scale Bar: 5um. (E) Quantification of (D), showing the number of RIF1 foci in each nucleus at each time point. More than 100 cells were picked. Results were presented as mean ± SEM. (F) Western blotting analysis with anti-53BP1 and anti-GAPDH antibodies in middle passage normal and HGPS fibroblasts. (TIF) [file pone.0167454.s004.tif]

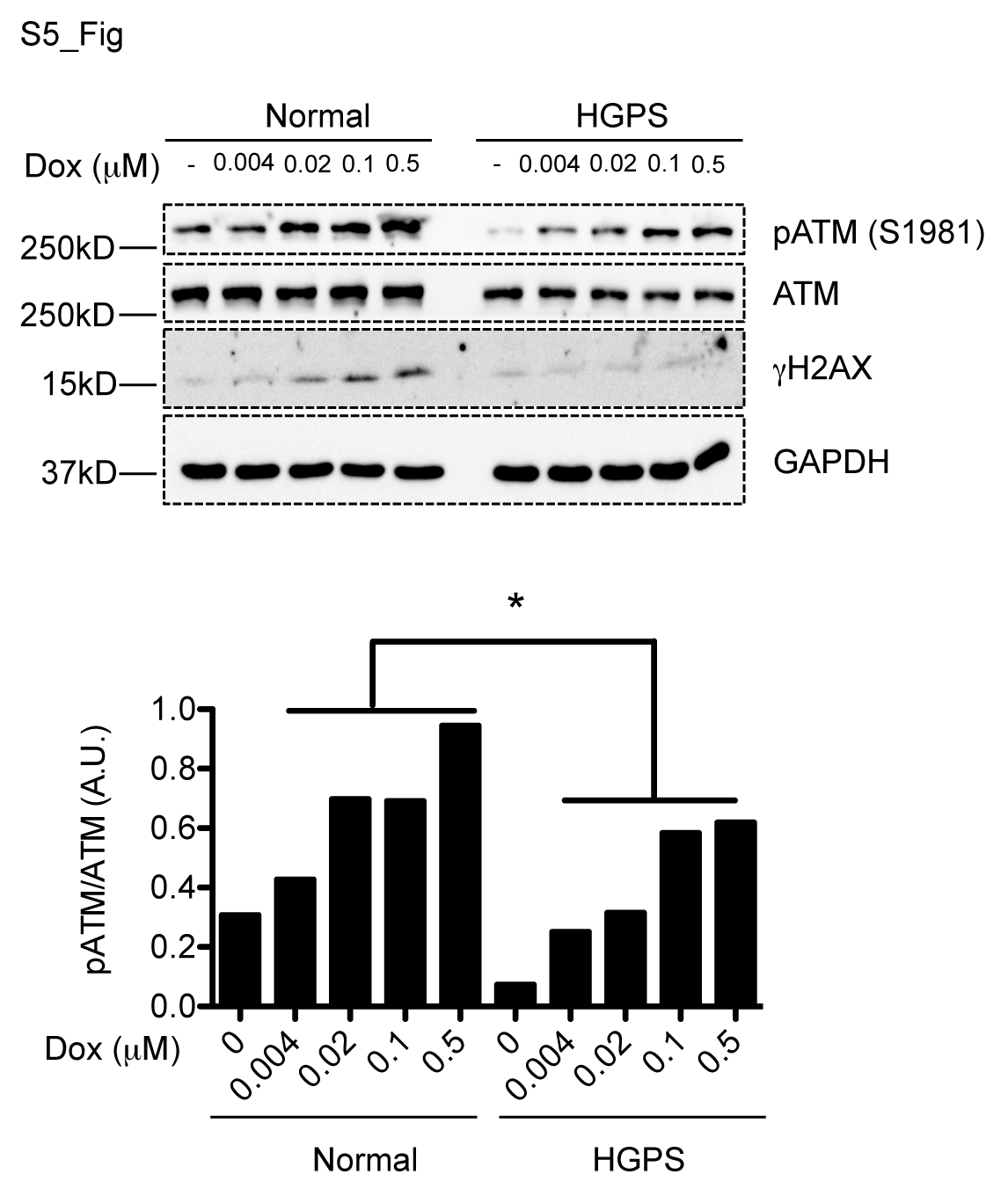

Supplement: S5 Fig — Western blotting analysis with anti-pATM(S1981), anti-ATM, anti- gammaH2AX and anti-GAPDH antibodies in middle passage normal and HGPS fibroblasts treated with indicated concentrations of Dox. (TIF) [file pone.0167454.s005.tif]

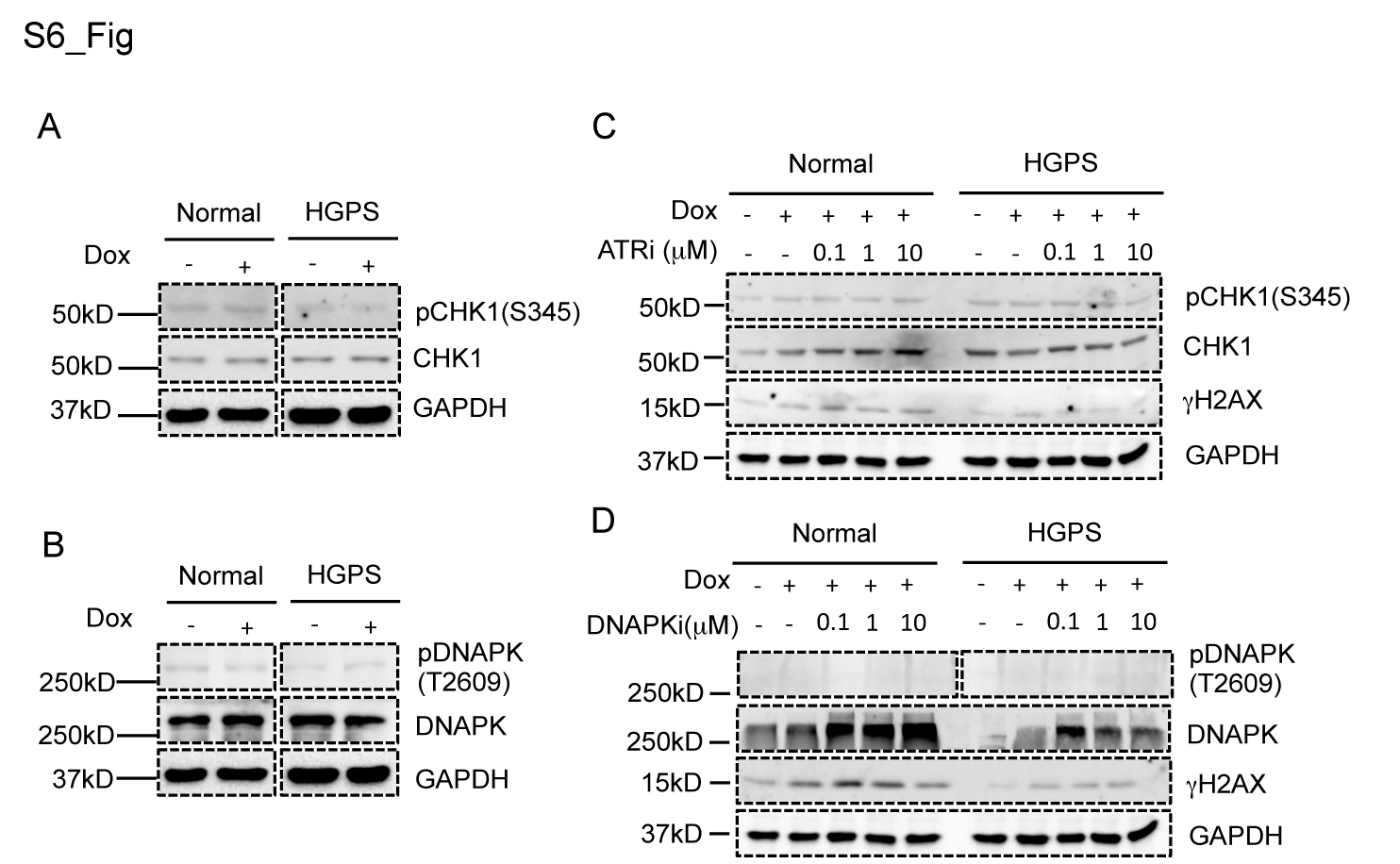

Supplement: S6 Fig — (A) Western blotting analysis with anti-pCHK1(S345), anti-CHK1 and anti-GAPDH antibodies on middle passage normal and HGPS fibroblasts after Dox treatment. ATR activation was indicated by phosphorylation of CHK1(S345). (B) Western blotting analysis with anti-pDNAPK(T2609), anti-DNAPK and anti-GAPDH antibodies on middle passage normal and HGPS fibroblasts after Dox treatment. DNAPK activation was indicated by phosphorylation of DNAPK(T2609). (C) Western blotting analysis with anti-pCHK1(S345), anti-CHK1, anti- gammaH2AX and anti-GAPDH antibodies on middle passage normal and HGPS fibroblasts pre-incubated with indicated concentrations of ATR inhibitor for 24h prior to Dox treatment. (D) Western blotting with anti-pDNAPK (T2609), anti-DNAPK, anti- gammaH2AX and anti-GAPDH antibodies on middle passage normal and HGPS fibroblasts pre-incubated with indicated concentrations of DNAPKcs inhibitor for 24h prior to Dox treatment. (TIF) [file pone.0167454.s006.tif]

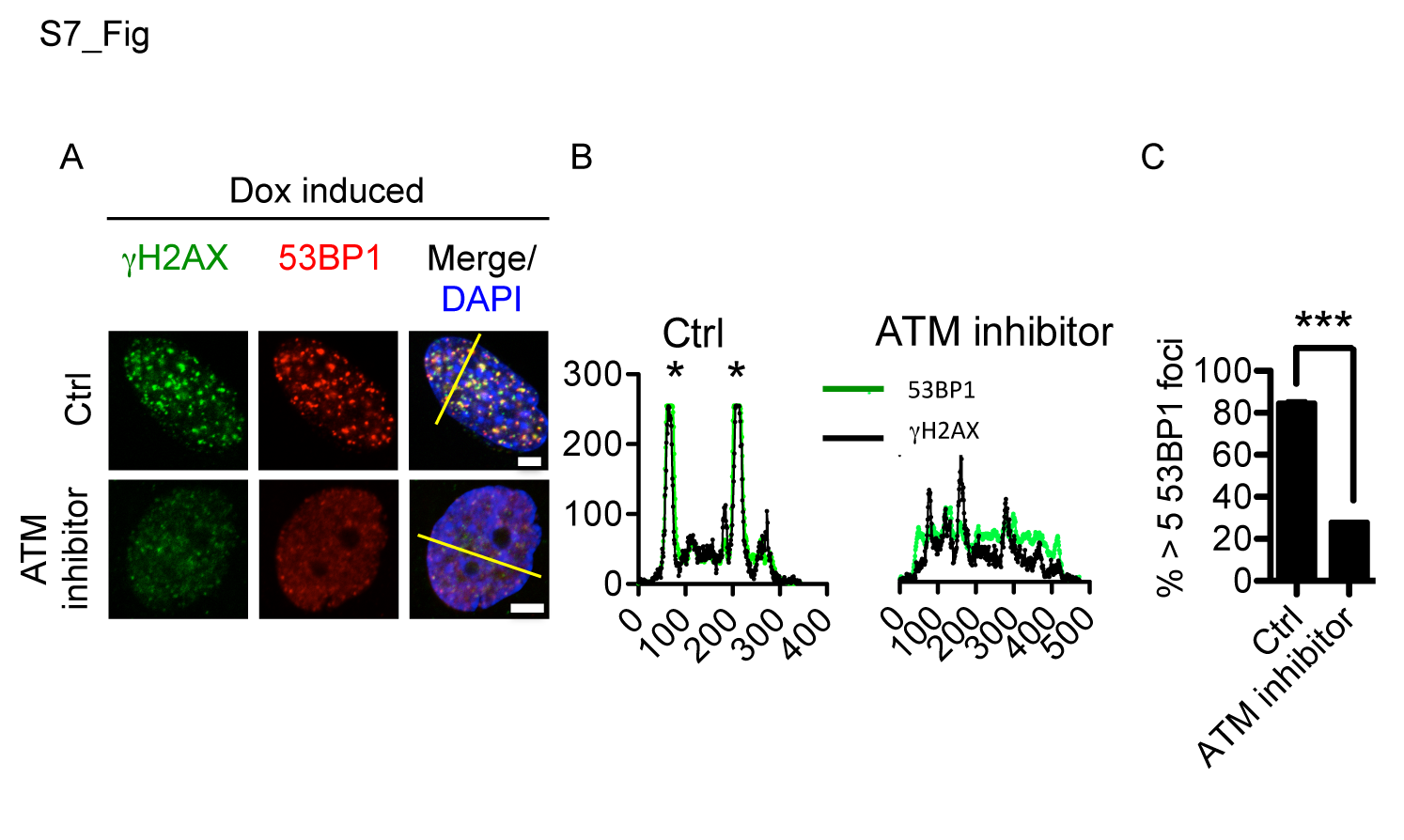

Supplement: S7 Fig — (A) Representative fluorescence images of gammaH2AX and 53BP1 in middle passage normal fibroblasts pre-incubated with or without 10uM ATM specific inhibitor prior to Dox treatment. Scale Bar: 5um. (B) Line profile analysis of (A), showing the reduced recruitment of 53BP1 to gammaH2AX foci in ATM inhibitor treated fibroblasts. Green (gammaH2AX) and red (53BP1) fluorescence intensities (y axis) were plotted against distance (x axis) along the yellow line in (A). Stars indicated strong co-localization of 53BP1 and gammaH2AX. (C) Quantification of the percentage of a population that displayed more than five 53BP1 foci in control or ATM inhibitor treated fibroblasts. Results were presented as mean ± SEM. ***P < 0.001. (TIF) [file pone.0167454.s007.tif]

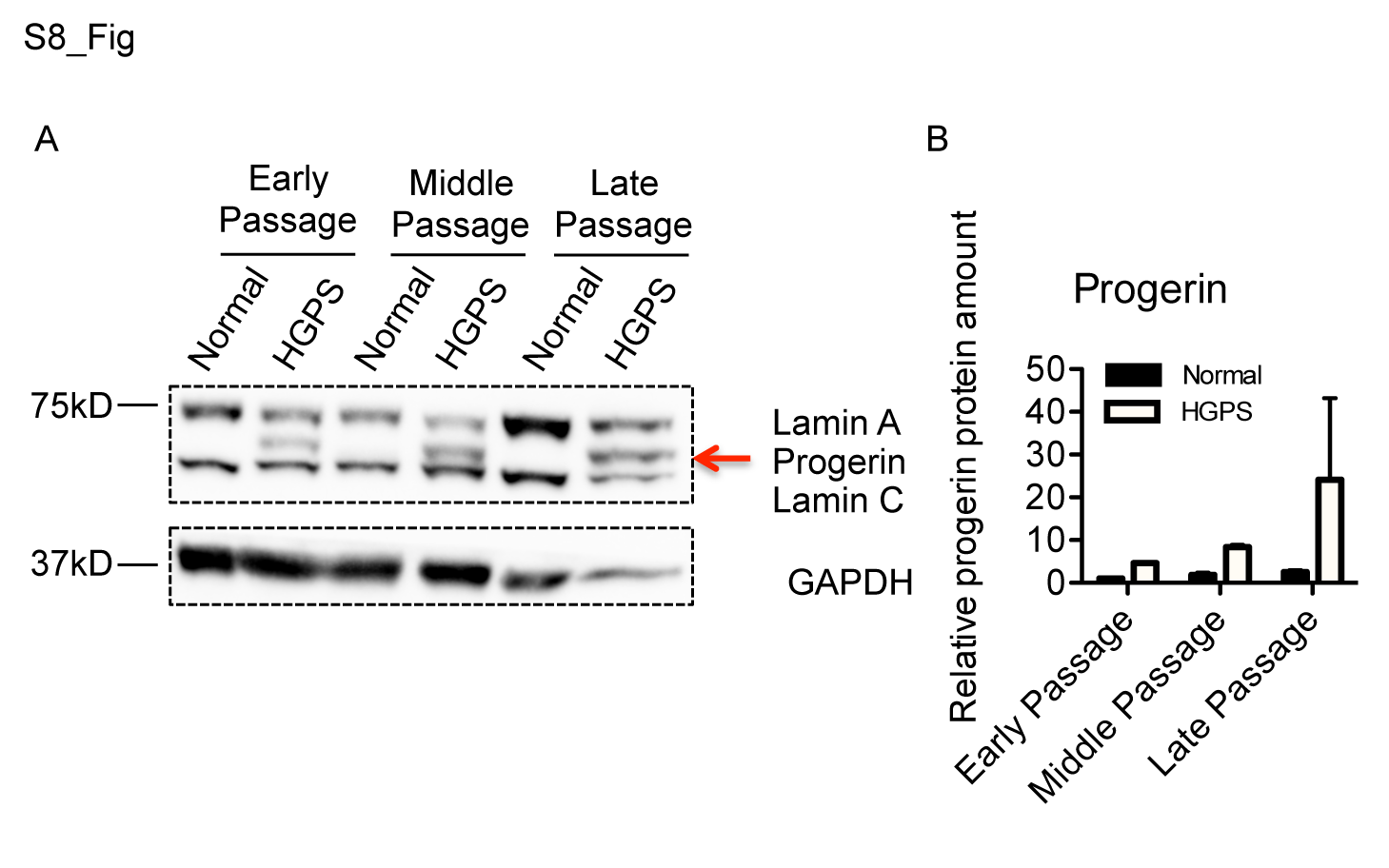

Supplement: S8 Fig — (A) Western blotting analysis with anti-Lamin A/C and anti-GAPDH antibodies on normal and HGPS fibroblasts at early, middle and late passages, showing the passage dependent accumulation of progerin in HGPS. (B) Quantification of (A), showing the relative band intensity of progerin (normalized to GAPDH) in early, middle and late passage HGPS fibroblasts. (TIF) [file pone.0167454.s008.tif]

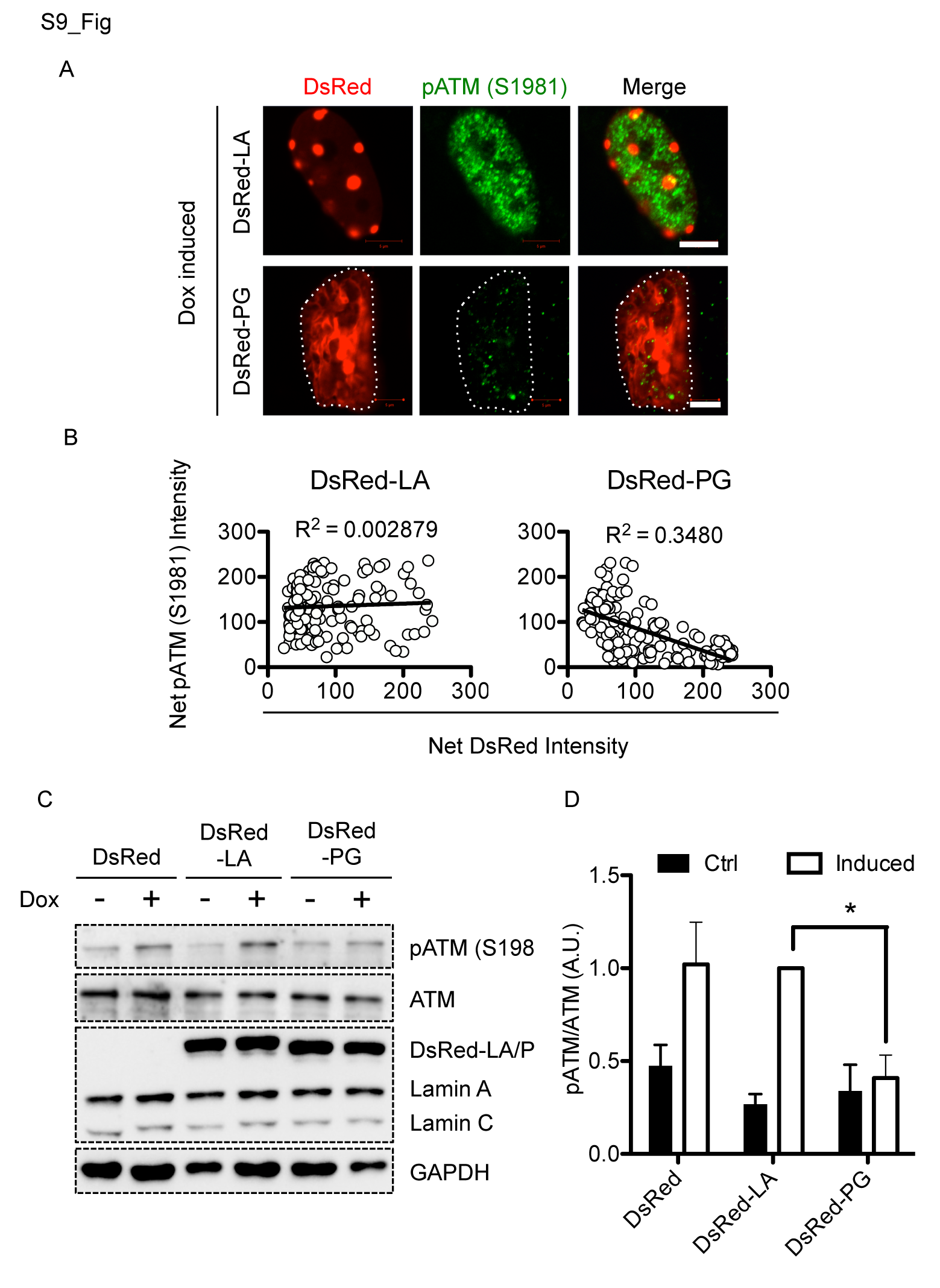

Supplement: S9 Fig — (A) Representative fluorescence images of Dox induced pATM(S1981) in late passage normal fibroblasts ectopically expressing DsRed, DsRed-lamin A (DsRed-LA) and DsRed-progerin (DsRed-PG) respectively. Scale Bar: 5um. (B) Quantification of (A), showing the fluorescence intensities of DsRed (x axis) and pATM(S1981) (y axis) in either DsRed-LA or DsRed-PG over expressing cells, after Dox treatment. Trend line and R2 were calculated using linear regression function. (C) Western blotting analysis with anti-pATM(S1981), anti-ATM, anti-lamin A/C and anti-GAPHD antibodies on late passage normal fibroblasts ectopically expressing DsRed, DsRed-lamin A (DsRed-LA) or DsRed-progerin (DsRed-PG). Cells were treated with or without Dox to induce DSBs before analysis. (D) Quantification of (C), showing the relative band intensity of pATM over total ATM before or after Dox treatment. Three independent experiments were performed. Results were presented as mean ± SEM. *P < 0.05. (TIF) [file pone.0167454.s009.tif]

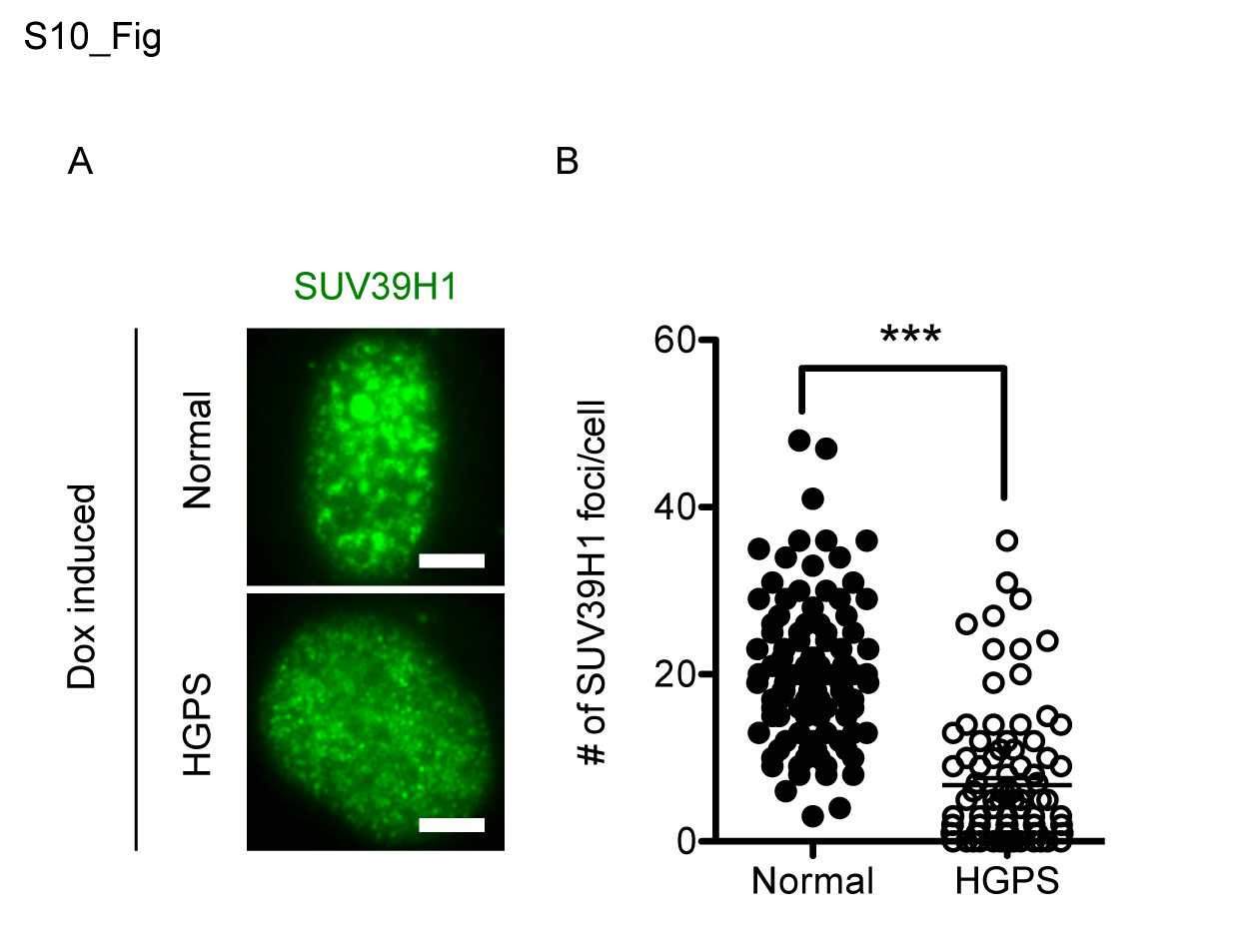

Supplement: S10 Fig — (A) Representative fluorescence images of Dox induced SUV39H1 foci formation in middle passage normal and HGPS fibroblasts. Scale Bar: 5um. (B) Quantification of (A), showing the number of SUV39H1 foci in each cell after Dox treatment. More than 100cells were randomly picked for each group. Results were presented as mean ± SEM. ***P < 0.001. (TIF) [file pone.0167454.s010.tif]

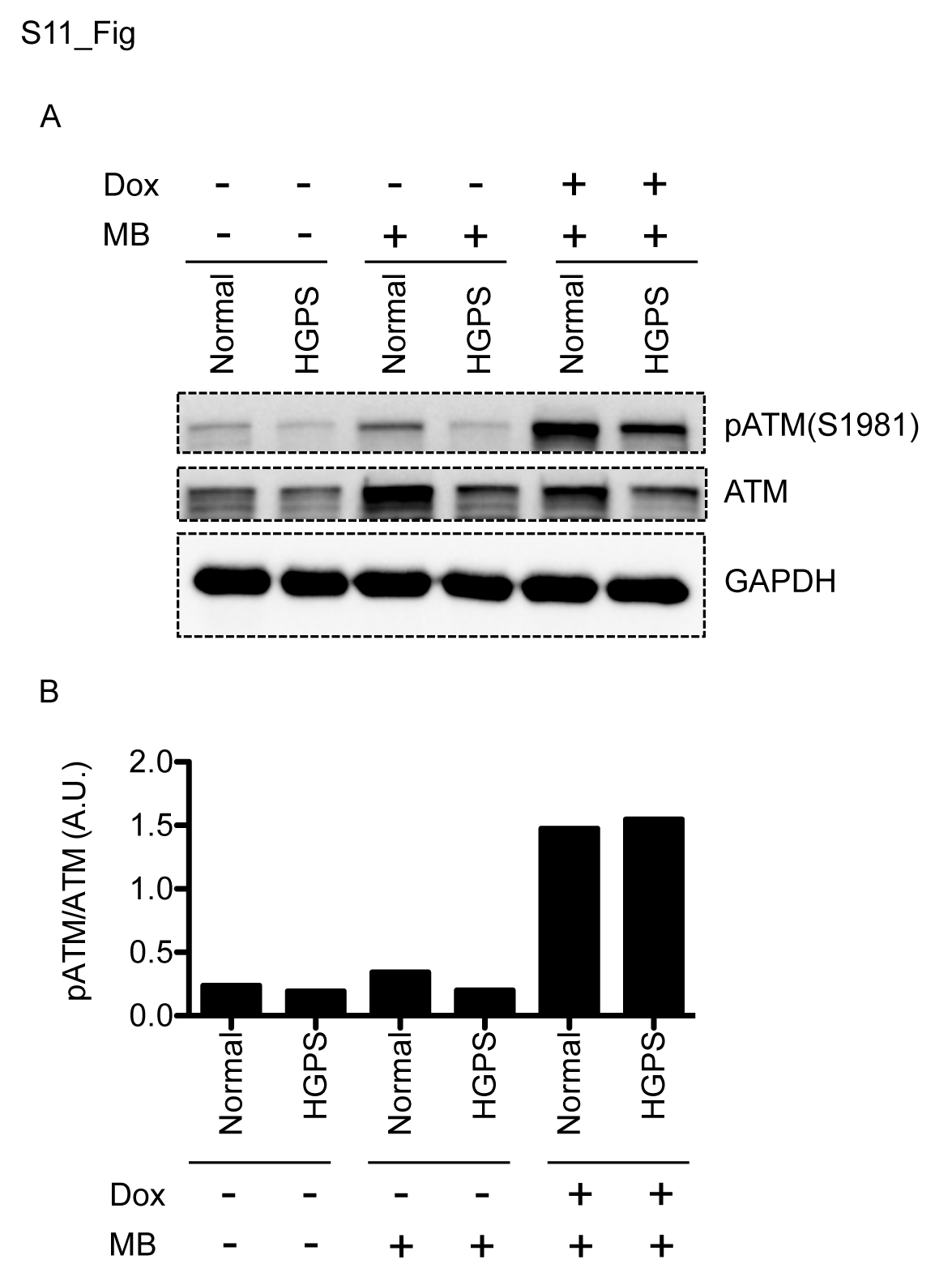

Supplement: S11 Fig — (A) Western blotting analysis with anti-pATM(S1981), anti-ATM and anti-GAPDH antibodies on late passage normal and HGPS fibroblasts with indicated treatments. (B) Quantification of (A), showing that the relative band intensity of pATM(S1981) over total ATM was unchanged at basal level (without Dox induction) in HGPS cells with/out methylene blue treatment. (TIF) [file pone.0167454.s011.tif]

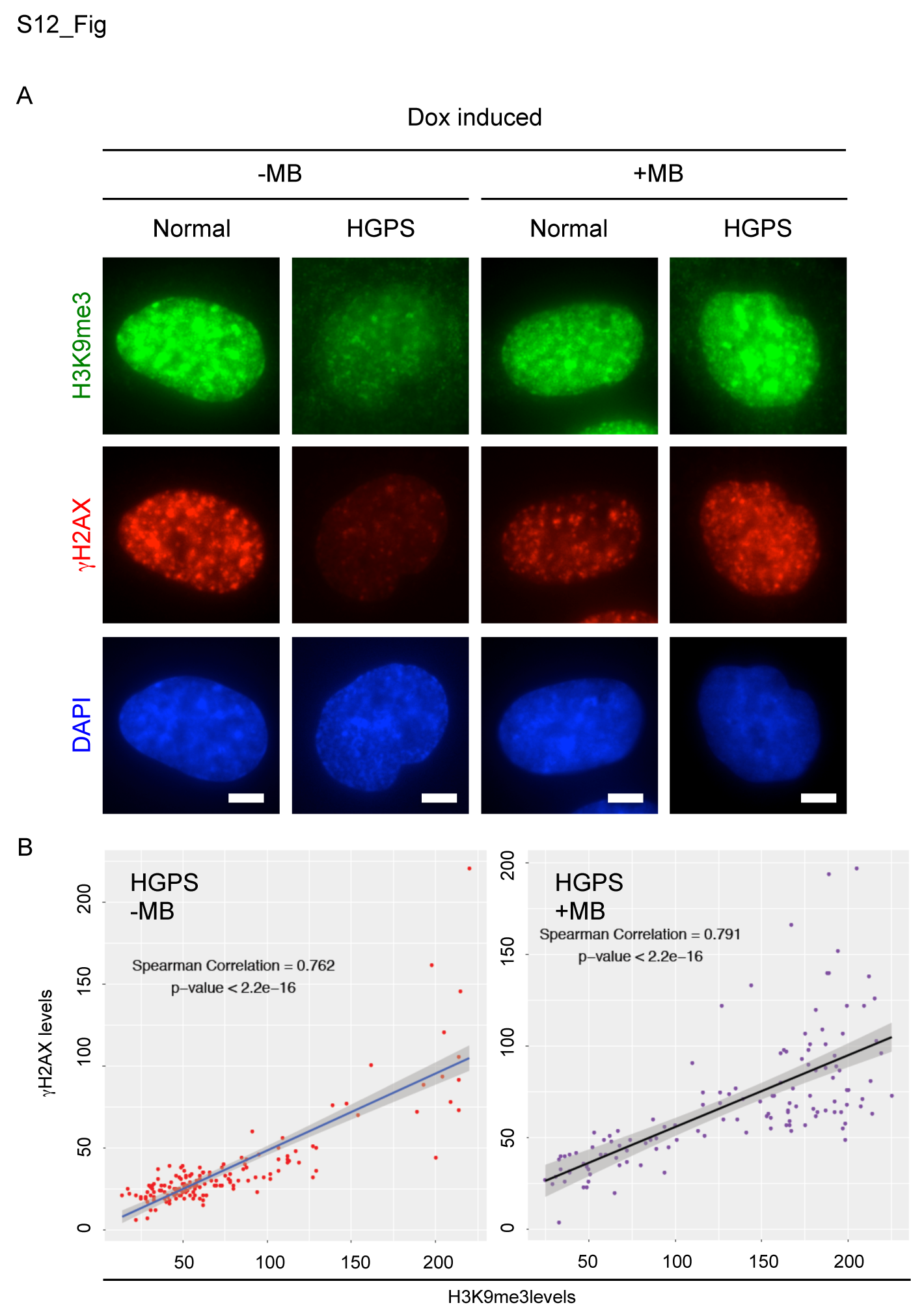

Supplement: S12 Fig — (A) Representative fluorescence images of H3K9me3 and gammaH2AX responses in late passage normal and HGPS fibroblast with or without methylene blue treatment. Scale Bar: 5um. (B) Quantification of (A), showing the Spearman correlations between H3K9me3 green fluorescence intensity and gammaH2AX red fluorescence intensity in HGPS fibroblasts with or without methylene blue treatment. More than 100 cells were randomly picked for quantification in each group. (TIF) [file pone.0167454.s012.tif]
